# In Vitro Antifungal and Antivirulence Activities of Biologically Synthesized Ethanolic Extract of Propolis-Loaded PLGA Nanoparticles against *Candida albicans*

**DOI:** 10.1155/2019/3715481

**Published:** 2019-11-30

**Authors:** Anupon Iadnut, Ketsaya Mamoon, Patcharin Thammasit, Sudjai Pawichai, Singkome Tima, Kanya Preechasuth, Thida Kaewkod, Yingmanee Tragoolpua, Khajornsak Tragoolpua

**Affiliations:** ^1^Division of Clinical Microbiology, Department of Medical Technology, Faculty of Associated Medical Sciences, Chiang Mai University, Chiang Mai 50200, Thailand; ^2^The Graduate School, Chiang Mai University, Chiang Mai 50200, Thailand; ^3^Division of Clinical Microscopy, Department of Medical Technology, Faculty of Associated Medical Sciences, Chiang Mai University, Chiang Mai 50200, Thailand; ^4^Infectious Diseases Research Unit (IDRU), Faculty of Associated Medical Sciences, Chiang Mai University, Chiang Mai 50200, Thailand; ^5^Department of Biology, Faculty of Sciences, Chiang Mai University, Chiang Mai 50200, Thailand

## Abstract

Propolis is a natural substance and consists of bioactive compounds, which gives it antioxidant and antimicrobial properties. However, the use of propolis is limited by the low solubility in aqueous solutions. Thus, nanoparticles may be likely to accomplish enhanced delivery of poorly water-soluble phytomedicine. The aim of the present study was to fabricate and evaluate the biological activity of ethanolic extract of propolis-loaded poly(lactic-co-glycolic acid) nanoparticles (EEP-NPs). The EEP-NPs were prepared using the oil-in-water (o/w) single-emulsion solvent evaporation technique. The physicochemical properties of EEP-NPs were characterized and tested on their cytotoxicity, antifungal activity, and impact on key virulence factors that contribute to pathogenesis of *C. albicans.* EEP-NPs were successfully synthesized and demonstrated higher antifungal activity than EEP in free form. Moreover, EEP-NPs exhibited less cytotoxicity on Vero cells and suppressed the virulence factors of *C. albicans*, including adhesion, hyphal germination, biofilm formation, and invasion. Importantly, EEP-NPs exhibited a statistical decrease in the expression of hyphal adhesion-related genes, *ALS3* and *HWP1*, of *C. albicans*. The results of this study revealed that EEP-NPs mediates a potent anticandidal activity and key virulence factors by reducing the gene-encoding virulence-associated hyphal- adhesion proteins of *C. albicans* and, thereby, disrupting the morphologic presence and attenuating their virulence.

## 1. Introduction

In recent times, natural products have become alternative therapeutic options because they are safe and effective with many active compounds [1]. Propolis is one of the most natural products that has been used as a traditional medicine. It is a natural resinous substance collected by bees from buds and exudates of plants, mixed with bee enzymes, pollen, and wax to seal cracks and crevices in their hive [2]. The propolis components depend on botanic and geographic origins and bee species [3]. In the past decade, several studies reported the properties of propolis on the antimicrobial activity [4–6], antiproliferation of cancer [7], antioxidant [8], and stimulation of immune function [9]. Although propolis is widely used in many applications as described above, the solubility of poorly soluble active compounds has been a limitation [10].

Currently, nanotechnology is applied in life sciences, especially the nanoparticles as a drug delivery system [11]. Advances in this system have led to the development of several aspects such as improved drug efficacy for infection diseases [12], targeted delivery for cancer therapy [13], and in cosmetics [14]. In particular, nanotechnology may be likely to accomplish enhanced delivery of poorly water-soluble phytomedicine [15].

Polymeric nanoparticles (PNPs) are one of the smart drug delivery platforms [16]. Many materials such as natural or synthetic polymers are needed to formulate PNPs [17]. PNP preparation has been reviewed elsewhere [18]. Poly(lactic-co-glycolic acid) (PLGA) is one of the most synthetic polymers for elaborating PNPs because it has a biodegradable property and has been approved by the Food and Drug Administration (FDA) for drug delivery [19]. Moreover, PLGA-based nanoparticles have been reviewed for various biomedical applications [20]. Therefore, propolis loaded into PLGA nanoparticles may overcome the limitation of water solubility and easily dispersed in aqueous media.

In this study, ethanolic extract of propolis-loaded PLGA nanoparticles (EEP-NPs) were formulated and characterized for the physicochemical properties. Then, the biological activities were evaluated for cytotoxicity and inhibitory effect on the growth of pathogenic yeast *C. albicans.* The virulence factors of yeasts, including adhesion, hyphal germination, biofilm formation, and invasion abilities, were also investigated. Moreover, the adhesion hyphal-related genes were examined using real-time RT-PCR. This study has importantly gained new preparation method for EEP-NPs and their efficacy on inhibiting *C. albicans* growth and virulence factors.

## 2. Materials and Methods

### 2.1. Propolis, Chemicals, and Reagents

Ethanolic extract of propolis (EEP) was kindly provided from the Bee Products Industry (Lamphun, Thailand). Poly(lactic-co-glycolic acid) (PLGA) (lactide : glycolide = 50 : 50; inherent viscosity 0.45–0.60 dl/g, Mw = 38–54 kDa) was purchased from Sigma-Aldrich (St. Louis, MO). Polyvinyl alcohol (PVA) and ethanol (EtOH) were purchased from Fluka (Buchs, Switzerland) and Merck Millipore (Darmstadt, Germany), respectively. Dichloromethane (DCM) was obtained from RCI Labscan (Gliwice, Poland). All the other chemicals and reagents used in this study were of analytical and molecular grade.

### 2.2. High-Performance Liquid Chromatography (HPLC) Analysis

The EEP was injected in HPLC equipment (Agilent 1100 Series, CA) and separated with an Agilent ZORBAX Eclipse XDB-C18 column; 4.6 × 150 mm, 5 *μ*m chromatographic column. The mobile phases were (A) 0.1% formic acid in water and (B) methanol. Separations were performed at room temperature by linear gradient elution from 0 min at 50% A/50% B to 35 min at 75% A/25% B with a flow rate of 1.0 ml/min. The eluent was continuously monitored at a wavelength of 267 nm for 35 min. The identification of the phenolic compounds was carried out by comparing the retention time of the sample with gallic acid, quercetin, pinocembrin, chrysin, and galangin standards. All analyses were performed in triplicate.

### 2.3. Preparation and Characterization of Ethanolic Extract of Propolis-Loaded PLGA Nanoparticles (EEP-NPs)

#### 2.3.1. Preparation of EEP-NPs

Three formulations of EEP-NPs were prepared using PLGA matrix (EEP-NP 1, EEP-NP 2, and EEP-NP 3) and three formulations of polymer control nanoparticles (nanoparticles without EEP; polymer-NP 1, polymer-NP 2, and polymer-NP 3) were also prepared. The suspensions of nanoparticles (NPs) were prepared by the modified oil-in-water (o/w) single-emulsion solvent evaporation method [21]. In brief, the organic solution consisting of EEP and PLGA were dissolved in absolute ethanol and dichloromethane (DCM), respectively. The ratios of EEP and PLGA (1 : 2, 1 : 1, and 2 : 1 for EEP-NP 1, EEP-NP 2, and EEP-NP 3, respectively) were mixed at room temperature until all materials were completely dissolved. Each organic solution was emulsified into 4 ml polyvinyl alcohol (2% w/v, PVA) in a dropwise manner. The resulting solution was stirred and then sonicated (output power 70 W, 2 min) using an ultrasonic processor UP100H (Hielscher Ultrasonics, Germany) within an ice bath. Each mixed solution was incubated overnight at room temperature in dark condition for complete polymerization. The resulting nanoparticles were centrifuged at 8,800*g* for 40 min at 4°C (Beckman Coulter, CA), washed once with deionized water, and then lyophilized. The three formulations of polymer control nanoparticles (polymer-NPs) were prepared with a similar method. All nanoparticles were stored at −20°C until used. The parameters for preparation of EEP-NPs and polymer-NPs are listed in Supplementary [Supplementary-material supplementary-material-1].

#### 2.3.2. Physicochemical Property Characterization of NPs

Dynamic light scattering (DLS) technique was used for determining the mean particle size and polydispersity index (PDI) values of EEP-NPs and polymer-NPs using a Zetasizer instrument (Malvern, UK) equipped with a 4.0 mV He-Ne laser (633 nm) [21]. Measurements were carried out in triplicate at 25 ± 0.1°C with using 0.8872 cP of viscosity. The number of runs and run time durations were chosen automatically. Zeta potential value of EEP-NPs and polymer-NPs was determined by the electrophoretic light scattering (ELS) technique and processed in a clear disposable zeta cell at 25 ± 0.1°C. All nanoparticle samples were diluted 1 : 10 with deionized water before measurement. Each sample was measured in triplicate, using 0.8872 cP for viscosity and 78.5 for dielectric constant. The measurement durations and voltage selections were set to automatic mode.

#### 2.3.3. Scanning Electron Microscopy (SEM)

The morphology of nanoparticles was observed using scanning electron microscopy (SEM). The samples were diluted 10-fold with deionized water, dropped onto stubs with double-sided copper tape, and dried in the desiccator overnight. Then, the samples were coated with gold film by using a JEOL JFC-1100E Ion Sputtering device (JEOL, Japan) under vacuum for 2 min and analyzed with a scanning electron microscope (SEM) (JEOL, Japan).

#### 2.3.4. Encapsulation Efficiency (EE) and Loading Capacity (LC)

The encapsulated EEP of nanoparticles was determined by an indirect quantification method by using UV-Vis spectrophotometer [21]. The supernatant of EEP-NPs was obtained after centrifugation at 8,800*g* for 40 min at 4°C. The supernatant was then diluted 1 : 2 with deionized water and the UV spectrum wavelength scanned over the range 258–400 nm by a UV-Vis spectrophotometer (Shimadzu, Columbia, MD). The EEP standard calibration curve was constructed by dissolved various concentrations of EEP in absolute ethanol. The amount of EEP in supernatant of EEP-NPs was calculated with the EEP standard calibration curve obtained by using the peak area. The EE and LC of EEP-NPs were quantified using the following equations [22]:

(1) $$\begin{array}{c} \text{EE}\left(\%\right)=\left(\frac{\text{amount of EEP encapsulated in NPs}}{\text{initial EEP added}}\right)\times 100,\\ \text{LC}\left(\%\right)=\left(\frac{\text{amount of EEP in NPs}}{\text{total amount of NPs}}\right)\times 100. \end{array}$$

#### 2.3.5. In Vitro Release Assay

The EEP release from nanoparticles was performed using a modified dissolution method [21]. One milligram of EEP-NPs was dissolved in 1 ml of phosphate buffer saline solution (PBS, pH 7.4) and shaken in an incubator at 37°C. The samples were collected at difference times (1, 2, 3, 6, 12, 18, and 24 h) and centrifuged at 8,800*g* for 20 min at 4°C. The supernatant was harvested and the pellet resuspended with 1 ml of fresh PBS. The release of EEP from the nanoparticles in the supernatant at different time points was measured by UV-Vis spectrophotometer over the spectral wavelength range as described above. The percentage of EEP released from the nanoparticles (% EEP release) was compared with EEP standard calibration curve.

### 2.4. Biological Properties of EEP-NPs

#### 2.4.1. Cytotoxicity Assay

Vero cells (African green monkey kidney epithelium) were maintained in Dulbecco's Modified Eagle's medium (DMEM) (Invitrogen, Green Island, NY) supplemented with 10% fetal bovine serum (FBS), penicillin (10 units/ml), and streptomycin (100 *μ*g/ml) in ambient conditions of 37°C and 5% CO_2_. For the cytotoxicity assay, 1 × 10^4^ cells were seeded in a 96-well plate and allowed to attach overnight. On the following day, five different concentrations of EEP-NPs and polymer-NPs were added and incubated at 37°C with 5% CO_2_ for 24 h. Twenty microliters of 5 mg/ml MTT (3-[4,5-dimethylthiazol-2-yl]-2,5 diphenyltetrazolium bromide) solution was added into each well and incubated for 4 h. One hundred microliters of dimethyl sulfoxide (DMSO) was added to each well to solubilize MTT formazan product. The optical density (OD) was measured at 570 nm using a microplate reader (BioTek, VT). The percentage of cell viability was quantified and compared with a cell control, which was a cell culture in media without NPs.

#### 2.4.2. Antifungal Assay

*C. albicans* DMST 21424, a biofilm producing strain, was obtained from the Department of Medical Science, Ministry of Public Health (MOPH), Thailand [23]. The yeast was cultured on Sabouraud dextrose agar (SDA) (HiMedia, Mumbai, India) at 37°C for 48 h. After the time of incubation, a single colony was picked into Sabouraud dextrose broth (SDB) (HiMedia, Mumbai, India) and incubated at 37°C overnight. The cells were harvested by centrifugation, resuspended with Roswell Park Memorial Institute (RPMI) 1640 (Gibco, Gaithersburg, MD), and adjusted to 1 × 10^4^ cells/ml. The antifungal activity against *C. albicans* at an equal amount of EEP in free form and EEP-NPs was investigated in cell viability and growth.

Various concentrations of EEP in free form and EEP-NPs were added into a 96-well tissue culture plate. One hundred microliters of 1 × 10^4^ cells/ml of *C. albicans* was then inoculated and incubated at 37°C overnight. The following day, the medium was removed and 50 *μ*l of 2 mg/ml MTT solution was added into each well and incubated at 37°C for 5 h. Two hundred microliters of DMSO was added into each well and the optical density at 540 nm was measured using a microplate reader.

The percentage of metabolic activity of EEP-NP-treated *C. albicans* was quantified and compared with the EEP in free form-treated *C. albicans*. To confirm the effect of EEP-NPs and EEP in free form on cell growth of *C. albicans*, the colony-forming unit (CFU) assay was performed. Yeast cells from respective wells were properly diluted and spread on SDA plates. The plates were incubated at 37°C for 24 h and counted, and the CFUs of yeast cells were calculated.

### 2.5. Antivirulence Factors of *C. albicans*

#### 2.5.1. Adhesion

Adhesion ability of *C. albicans* was determined using fluorescence assay [24]. Vero cells (1 × 10^5^ cells) were grown in a 96-well black tissue culture plate and incubated at 37°C with 5% CO_2_. The yeast cells were labeled with 25 *μ*g/ml of Concanavalin AAlexa 488 (Invitrogen, NY) for 1 h in dark condition. Then, the labeled *C. albicans* were adjusted to 100 MOI and treated with various concentrations of EEP-NPs and polymer-NPs and inoculated into a 96-well black tissue culture plate that contained Vero cells. The cocultivated cells were incubated at 37°C with 5% CO_2_ for 90 min. Then, nonadherent labeled *C. albicans* cells were washed thrice with PBS. Mean fluorescence intensity (MFI) was measured using a microplate reader at excitation and emission wavelengths of 495/519 nm. The MFI of adherent yeasts was converted to the percentage of adhesion compared with control with the following formula:

(2) $$\begin{array}{c} \text{adhesion}\left(\%\right)=\left(\frac{{\text{MFI}}_{\text{test}}}{{\text{MFI}}_{\text{control}}}\right)\times 100, \end{array}$$

where MFI_test_ was the mean fluorescence intensity of the labeled yeast cells incubated with NP suspension and MFI_control_ was the mean fluorescence intensity of the labeled yeast cells incubated with culture medium only.

#### 2.5.2. Hyphal Germination

*C. albicans* (1 × 10^7^ cells) were inoculated into inducer media (10% FBS-RPMI 1640) and treated with various concentrations of EEP-NPs and polymer-NPs [25]. After 4 h incubation at 37°C, the hyphal form of yeast cells was observed under a light microscope. Five hundred yeast cells were counted, and the percentage of hyphal germination was calculated from the following formula:

(3) $$\begin{array}{c} \text{hyphal germination}\left(\%\right)=\left(\frac{\text{number of hyphal form}}{\text{number of total yeast cells}}\right)\times 100. \end{array}$$

#### 2.5.3. Biofilm Formation

*Candida* biofilm formation was determined by the XTT reduction assay [26]. In brief, overnight culture of *C. albicans* in SDB was centrifuged, washed, and adjusted to 1 × 10^7^ CFU/ml in RPMI-1640 medium. One hundred microliters of *C. albicans* was seeded into a 96-well polystyrene plate and incubated for 90 min at 37°C with 5% CO_2_. Nonadhered cells were removed by washing thrice with sterile PBS. Two hundred microliters of various concentrations of EEP-NPs and polymer-NPs were then added, mixed well, and incubated at 37°C for 24 h. After incubation, biofilms were washed thrice with PBS and the biofilm mass measured by the XTT dye [2,3-bis (2-methoxy-4-nitro-5-sulfophenyl)-2H-tetrazolium-5-carboxanilide sodium salt] (Invitrogen, NY). In the prior assay, one hundred microliters of 0.5 mg/ml XTT solution was mixed with 1 *μ*M menadione (Bio Basic Inc., Canada) and added into each well. After incubation for 1 h at 37°C, the colorimetric change of XTT reduction was measured by a microplate reader at 492 nm.

#### 2.5.4. Hyphal Adhesion-Related Gene Expression

Hyphal adhesion-related gene expression was investigated by real-time RT-PCR [27]. In brief, Vero cells (1 × 10^5^ cells) were grown in a 96-well tissue culture plate and incubated at 37°C with 5% CO_2_. After reaching ∼80% confluence, the cells were cocultured with *C. albicans* (100 MOI) and treated with various concentrations of EEP-NPs and polymer-NPs. The cocultivated cells were incubated at 37°C in 5% CO_2_ for 12 h. Then, nonadherent *Candida* cells were washed thrice with PBS. After that, trypsin-EDTA solution was added and the cell suspension harvested. Vero cells were lysed with diethyl pyrocarbonate- (DEPC-) treated water and yeast cells were collected. The yeast pellet was treated with solution/lysis buffer for 1 h at 30°C and then centrifuged at 1,000*g* for 5 min [28]. Spheroplast cells were extracted for total RNA with the NucleoSpin RNA II extraction kit (Macherey-Nagel GmbH & Co. KG, Düren, Germany) and reverse-transcribed with the RevertAid First Strand cDNA Synthesis kit (Thermo Scientific, Rockford, IL), according to the instructions of the manufacturer. Complementary DNA (cDNA) was amplified with specific primers (as shown in Supplementary [Supplementary-material supplementary-material-1]) by real-time RT-PCR. The reaction mixture contained 10 *μ*l of SensiFAST™ SYBR Green Master Mix (Bioline, Boston, MA), 300 nM of each primers and 2 *μ*l of cDNA sample in a total volume of 20 *μ*l. The PCR reactions were performed with initial denaturation at 95°C for 10 min, followed by 40 cycles of 15 s at 95°C, 30 s at 60°C, and 60 s at 72°C. The specific amplicon was detected by melting curve analysis. *ACT1* was used as a house keeping gene and the mRNA expression was normalized using the 2^−ΔΔCT^ method [29].

#### 2.5.5. Invasion

Invasion ability of *C. albicans* was detected by the cell damage assay [30]. In brief, Vero cells (5 × 10^4^ cells) were cultured in a 96-well tissue culture plate and incubated at 37°C in 5% CO_2_. The cells were cocultured with *C. albicans* (5 × 10^6^ cells), which were treated with various concentrations of EEP-NPs and polymer-NPs. The cocultivation was incubated for 48 h at 37°C in 5% CO_2_. Supernatants were collected and the lactate dehydrogenase (LDH) measured by using an LDH-cytotoxicity colorimetric assay kit (BioVision, CA). Samples containing only Vero cells or yeast cells alone were used as controls. Percentage of Vero cells damage was calculated by the following formula:

(4) $$\begin{array}{c} \text{cell damage}\left(\%\right)=\left(\frac{{\text{OD}}_{\text{Treatment}}-{\text{OD}}_{\text{Cell subtracted}}-{\text{OD}}_{\text{yeast subtracted}}}{{\text{OD}}_{\text{Control}}}\right)\times 100, \end{array}$$

where OD_Treatment_ was the optical density of coincubation between NP-treated yeast cells and Vero cells, OD_Cell subtracted_ was the optical density of NP-treated Vero cells, OD_yeast subtracted_ was the optical density of NP-treated yeast cells, and OD_Control_ was the optical density of coincubation between yeast cells and Vero cells.

The invasion of yeast cells was confirmed by a scanning electron microscope (SEM) [31]. In brief, a sterile cover glass (12 mm) was placed into a 24-well tissue culture plate and coated with 2 *μ*g/ml poly-lysine for 1 h at RT. Vero cells (1 × 10^5^ cells) were seeded into each well and incubated at 37°C for 24 h in 5% CO_2_. Then, the cells were cocultured with *C. albicans* (100 MOI), which were treated with various concentrations of EEP-NPs and polymer-NPs for 48 h at 37°C in 5% CO_2_. The media was removed, washed thrice with PBS, and fixed with 2.5% glutaraldehyde (pH 7.2) for 2 h at 4°C. Afterwards, the samples were washed thrice with PBS and postfixed with 2% osmium tetroxide (OsO_4_) for 2 h. All samples were dehydrated by stepwise ethanol gradients: 50% and 70% (twice for 10 min), and 95% (twice for 5 min) and 100% (twice for 1 min), respectively. Finally, the samples were coated with gold film using a JEOL JFC-1100E Ion Sputtering device (JEOL, Japan) under vacuum for 2 min and the cell morphology was determined using a scanning electron microscope (SEM).

### 2.6. Statistical Analysis

All data were represented as the mean ± standard error of the mean (SEM) of three independent experiments in triplicate. Significant differences (^*∗*^*p* < 0.05) when compared with the control group were determined by one-way ANOVA with the Tukey–Kramer multiple comparisons posttest.

## 3. Results

### 3.1. HPLC Analysis

**Figure 1:**
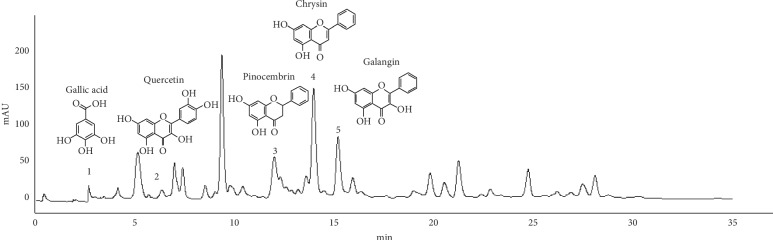
Chromatograms of EEP according to the quantification transitions of gallic acid, quercetin, pinocembrin, chrysin, and galangin.

HPLC chromatograms are presented in Figure 1. The analysis of the brown propolis revealed the presence of gallic acid, quercetin, pinocembrin, chrysin, and galangin (retention times: 2.66, 6.32, 12.32, 13.95, and 15.17 min, respectively).

### 3.2. Physicochemical Properties of NPs

**Figure 2:**
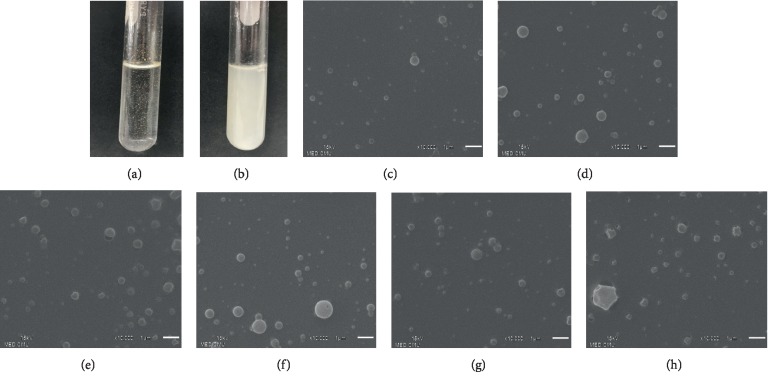
Characteristics of EEP-NPs. Comparative solubility of EEP powder and EEP-loaded PLGA nanoparticles (EEP-NPs). (a) EEP in water and (b) EEP-NPs in water. Scanning electron microscopy images of nanoparticles. (c) EEP-NP 1, (d) EEP-NP 2, (e) EEP-NP 3, (f) polymer-NP 1, (g) polymer-NP 2, and (h) polymer-NP 3. Scale bar represents 1 *μ*m and magnification 10,000×.

The EEP-NPs were successfully formulated and the water solubility was observed by visualization. The results showed EEP was incompletely dissolved in the water and exhibited the aggregation as shown in Figure 2(a). In particular, the lyophilized form of EEP-NPs exhibited a water-soluble property and presented the homogeneous solution (Figure 2(b)). The morphology of NPs was confirmed under a scanning electron microscope (SEM). All formulation of EEP-NPs and polymer-NPs exhibited a spherical shape with a smooth surface as shown in Figures 2(c)–2(h).

All formulations of NPs demonstrated mean particle size in diameter less than 500 nm by dynamic light scattering (DLS) analysis. These particle sizes indicated that all formulations are particles in nanometer scale. In addition, zeta potential was used to evaluate particles surface charge. The zeta potential values of all NP formulations were negative charge in the range between −1.2 ± 1.1 mV and −3.9 ± 0.5 mV. The PDI values of EEP-NP 1 and EEP-NP 2 represented 0.36 ± 0.02 and 0.57 ± 0.08, respectively. The PDI value of EEP-NP 3 was 0.73 ± 0.13. The percentage of encapsulation efficacy (EE) of all NP formulation represented approximately 90% of EE, which was successfully encapsulated into NPs. In addition, the percentage of loading capacity (LC) of EEP-NPs tended to increase in the proportion of EEP, which was 28.6 ± 1.1% of EEP-NP 1 to 56.7 ± 3.4% of EEP-NP 3. The summarized results of physicochemical properties of NPs are shown in Table 1.

### 3.3. In Vitro Release of Propolis from EEP-NPs

**Figure 3:**
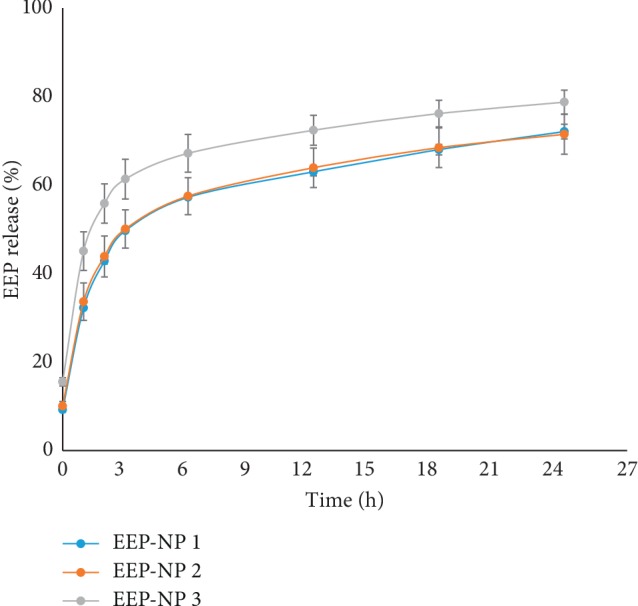
In vitro release profiles of EEP-NPs in PBS buffer (pH 7.4) at 37°C. Data are represented as mean ± SEM of three independent experiments performed in triplicate.

The EEP release from NPs was carried out in PBS in pH 7.4 at 37°C. The supernatant was aspirated, and the release profile of EEP from NPs was measured. As shown in Figure 3, all formulations of NPs showed a typical two-phase release pattern.

The initial burst release of EEP from EEP-NP 1, EEP-NP 2, and EEP-NP 3 was found in the first 6 h of the incubation period and was 57.3 ± 0.3%, 57.5 ± 4.2%, and 67.2 ± 4.3%, respectively. The second phase, referred to as the sustained release, was observed from 7 to 24 h, which was related to the diffusion mechanism and polymer chain cleavage. The sustained release reached 72.1 ± 1.7%, 71.5 ± 4.5%, and 78.8 ± 2.7% at 24 h for EEP-NP 1, EEP-NP 2, and EEP-NP 3, respectively. It was clearly seen that the percentage of EEP release in EEP-NP 3 was higher than that of EEP-NP 1 and EEP-NP 2 as shown in Figure 3. There were no difference in the percentage of EEP release in EEP-NP 1 and EEP-NP 2.

EEP-NP 1 and EEP-NP 2 were chosen for biological experiments due to the appropriate value of PDI and release profile. The lower PDI values of EEP-NP 1 and EEP-NP 2 were highly monodispersed than that of EEP-NP 3. In addition, the release profiles of EEP-NP 1 and EEP-NP 2 were lower than that of EEP-NP 3. The high burst release of EEP-NP 3 was not suitable for the drug release property of nanoparticles. This phenomenon might not reach to the target tissue or cells, thus resulting in loss of drug efficacy [20].

### 3.4. Cytotoxicity of EEP-NPs

**Figure 4:**
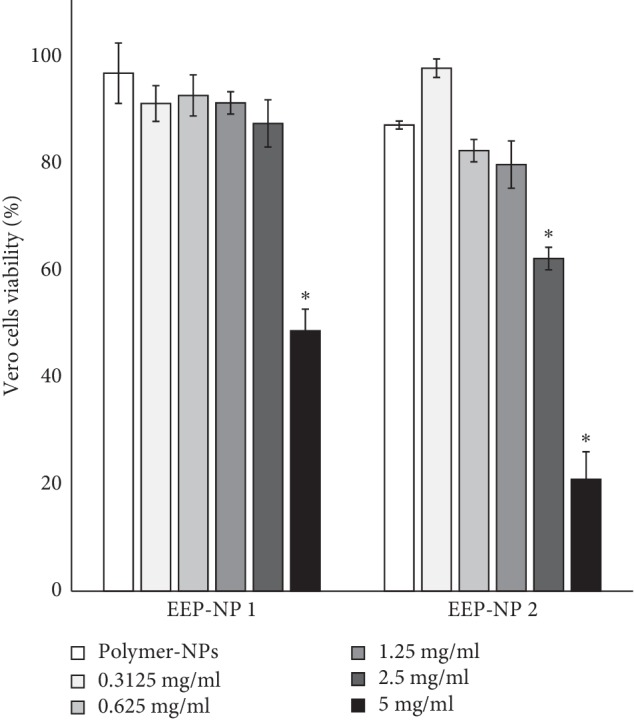
Cytotoxicity of EEP-NPs on Vero cells. Data are expressed as mean ± SEM of three independent experiments in triplicate. ^*∗*^*p* < 0.05 as determined by one-way analysis of variance.

The 50% cytotoxic concentration (CC_50_) of EEP-NPs on Vero cells was investigated and chosen for antifungal testing. The CC_50_ of EEP-NP 1 and EEP-NP 2 was less than 5 mg/ml and 2.5 mg/ml, respectively. On the contrary, both polymer-NPs had not affected the growth of Vero cells as shown in Figure 4.

### 3.5. Antifungal Activity

**Figure 5:**
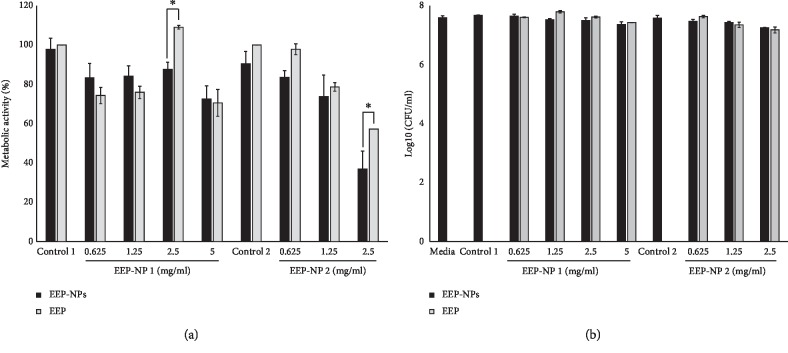
Determination of antifungal activity of EEP-NPs and EEP in free form. (a) MTT assay and (b) colony-forming unit assay. Data are expressed as mean ± SEM of three independent experiments in triplicate. ^*∗*^*p* < 0.05 as determined by one-way analysis of variance.

Antifungal activity of EEP in free form and EEP-NPs against *C. albicans* was investigated by MTT assay (Figure 5(a)) and colony-forming unit (CFU) assay (Figure 5(b)). Inhibitory effects of EEP-NP 1 on the metabolic activity of *C. albicans* were approximately 10% when compare with polymer control 1. However, the metabolic activity of EEP-NP 2-treated *C. albicans* reduced by dose-dependent manner. Interestingly, EEP-NP 2 at the concentration of 2.5 mg/ml had significantly decreased the metabolic activity of *C. albicans* approximately 60% when compared to polymer control 2. Both EEP-NP 1 and EEP-NP 2 at concentration of 2.5 mg/ml showed significantly reduced the metabolic activity of *C. albicans* approximately 20% when compare with the free form of EEP. However, all concentrations of EEP-NPs and EEP in free form-treated yeasts did not affect the growth of *C. albicans* in CFU assay (Figure 5(b)). We, therefore, conclude that EEP NP 2 has superior antimetabolic activity than EEP-NP 1, and it was chosen for further experiments.

### 3.6. Antivirulence Effects and Expression of Adhesion Hyphal-Related Genes of *C. albicans*

#### 3.6.1. Adhesion

**Figure 6:**
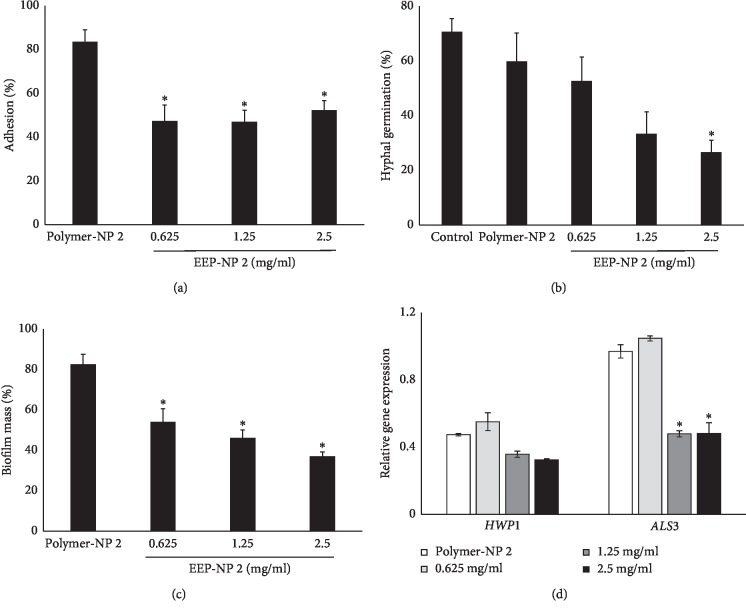
EEP-NP 2 reduced the key virulence factors of *C. albicans.* (a) Adhesion, (b) hyphal germination, and (c) biofilm formation. (d) Adhesion hyphal-related mRNA expression of *HWP1* and *ALS3* was determined and normalized to the *ACT1* mRNA level and expressed as a fold change with the control. Data are expressed as mean ± SEM of three independent experiments in triplicate. ^*∗*^*p* < 0.05 as determined by one-way analysis of variance.

Initially, we investigated whether EEP-NP 2 affects the adhesion ability of *C. albicans*, which is an important first step of *Candida* pathogenesis. The adherence of conA-Alexa Fluor 488-labeled *C. albicans* yeast cells to Vero cells and the mean fluorescence intensity (MFI) were measured, and the percentage of adhesion was calculated. The result showed that the adherence ability of EEP-NP 2-treated *C. albicans* to Vero cells was found to have significantly reduced approximately 40% at all concentrations compared with the growth and polymer-NP 2 controls as shown in Figure 6(a).

#### 3.6.2. Hyphal Germination

The ability of yeast-to-hyphal transition represents one of the key virulence factors associated with the pathogenesis of *C. albicans* infections. Therefore, the effect of EEP-NP 2 on hyphal germination of *C. albicans* was investigated. Results showed that the conversion to germ tubes was reduced in EEP-NP 2-treated *C. albicans* by a dose-dependent manner after 4 h of incubation (Supplementary Figure 1). The percentage of hyphal germination of EEP-NP-2 treated *C. albicans* tended to decrease approximately 52.6%, 33.4%, and 26.6%, at concentrations of 0.625, 1.25, and 2.5 mg/ml, respectively (Figure 6(b)). Furthermore, there was no significant difference in the percentage of hyphal germination between polymer-NP 2-treated and untreated *C. albicans*. These results indicated that PLGA was nontoxic to *C. albicans* and EEP-NP 2 contained some inhibitors of *C. albicans* morphogenesis.

#### 3.6.3. Biofilm Formation

A further important virulence factor of *C. albicans* is its ability to form biofilms. Mature biofilm was resistant to various antifungal agents [32]. To examine the effects of EEP-NP 2 on *C. albicans* biofilm formation, the biofilm metabolic activity was determined by XTT assay. The results showed that EEP-NP 2 significantly reduced *C. albicans* biofilm formation in a dose-dependent manner (Figure 6(c)). Specifically, EEP-NP 2 inhibited biofilm formation approximately by 34.6%, 44.1%, and 55.3% at concentrations of 0.625, 1.25, and 2.5 mg/ml, respectively, compared with polymer-NP 2 (Figure 6(c)).

#### 3.6.4. Adhesion Hyphal-Related Gene Expression

From the previous results, the EEP-NP 2 was able to functionally inhibit the adhesion of *C. albicans*. This effect leads to reduce biofilm formation of *C. albicans*. Transcriptional changes in adhesion and hyphal related-genes during the early phase of *Candida* pathogenesis were deeply investigated. The cDNA synthesis and real-time RT-PCR were performed for determination agglutinin-like sequence 3 (*ALS3*) and hyphal wall protein 1 (*HWP1*) genes expression. The results showed that *HWP1* mRNA expression was slightly downregulated by the dose-dependent characteristic of EEP-NP 2. Interestingly, the *ALS3* mRNA expression was remarkably decreased at 2.0-fold changes at concentrations of 1.25 and 2.5 mg/ml (Figure 6(d)).

#### 3.6.5. Invasion

**Figure 7:**
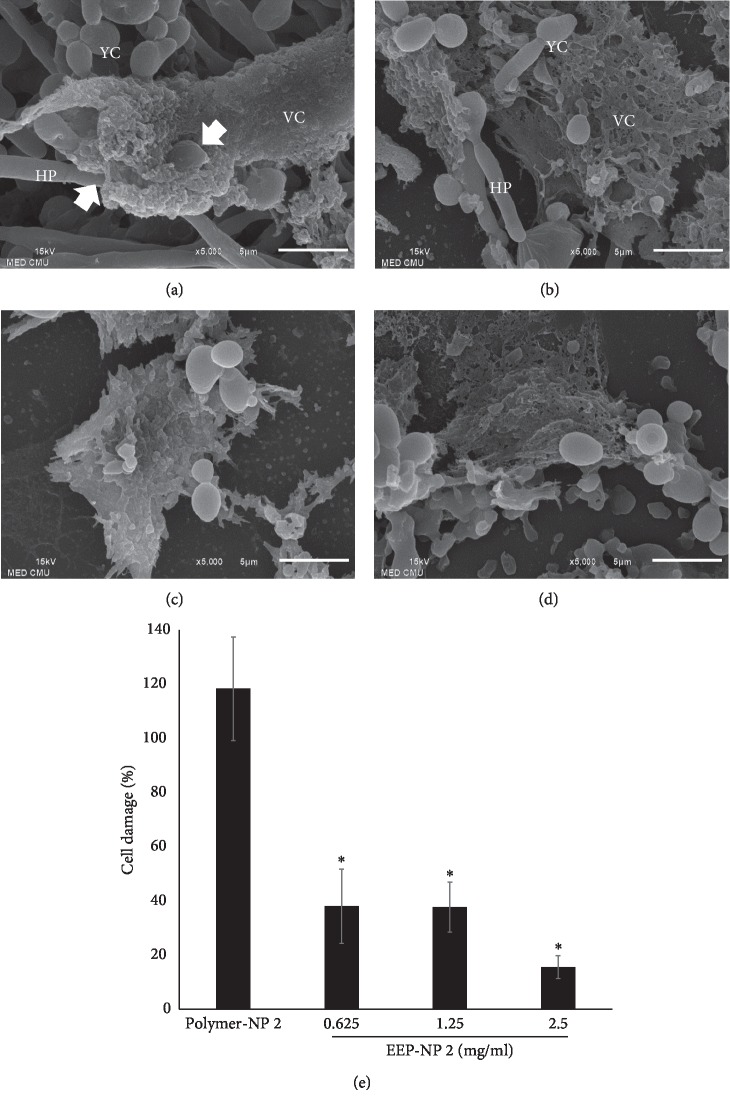
EEP-NP 2 inhibited *C. albicans* invasion. The yeast cells were treated with (a) polymer-NP 2, (b) 0.625, (c) 1.25, and (d) 2.5 mg/ml of EEP-NP 2 and cocultured with Vero cells and imaged by a scanning electron microscope (SEM) with a magnification of 5,000×. White arrows represent the location of yeast penetration into Vero cells. VC: Vero cells; YC: yeast cells; HP: hyphae. Scale bar represents 5 *μ*m. (e) Cell damage assay was quantified by LDH release from Vero cells. Data are represented as mean ± SEM of three independent experiments performed in triplicate. ^*∗*^*p* < 0.05 as determined by one-way analysis of variance.

Besides adhesion and hyphal germination abilities, invasion of *C. albicans* is also an important step in the physiopathology of Candidiasis. Therefore, the effect of EEP-NP 2 on the invasion of *C. albicans* has been observed by a scanning electron microscope (SEM). The results showed that polymer-NP 2-treated *C. albicans* had successfully possessed the ability to germinate with long filamentous hyphae and actively penetrated into Vero cells, resulting in cell damage (Figure 7(a)). Short hyphae and yeast cells were observed in EEP-NP 2-treated *C. albicans* at concentrations of 0.625 mg/ml and also adhered to the Vero cells (Figures 7(b) and 7(c)). Notably, *C. albicans* existed in planktonic cells when treated with EEP-NP 2 at a concentration of 1.25 and 2.5 mg/ml and reduced the ability to damage the Vero cells (Figure 7(d)).

In addition, the cell damage by *C. albicans* was confirmed using LDH release assay. The releasing of LDH from Vero cells was markedly decreased in *C. albicans* treated with all concentrations of EEP-NP 2 when compared with polymer-NP 2, as shown in Figure 7(e). These exhibited the inhibitory ability to invade epithelial cells of *C. albicans* and resulting in reduction of cell damage.

## 4. Discussion

Currently, problems with the empiric regimen, including inappropriate use and a delayed treatment, might be caused by the rapid and extensive emergence of antimicrobial resistance or more toxic effects to patients [33]. Natural products have played a very important role in health care and prevention of diseases. Ethanolic extract of propolis (EEP) is one of the most natural products that has antimicrobial activity. However, the variation in antimicrobial potential of propolis is linked to a particular chemical composition due to the different continents, regions, and plant species used to produce propolis by honey bees [34]. More than 300 different components have been identified in propolis in general, polyphenol (flavonoids, phenolic acids, and esters), phenolic aldehydes, ketones, etc. [35–37]. The chemical analysis of the brown propolis, herein, revealed the presence of flavones (chrysin), flavanones (pinocembrin), and flavonols (galangin) being the most common brown propolis components as described elsewhere [38–40]. EEP not only has a potential antifungal activity to inhibit the growth of *C. albicans* and non-*albicans Candida* (NAC) species [41] but also exhibited an inhibitory effect on the biofilm formation of *C. albicans* [42]. However, the utilization of EEP has been limited to its poor water solubility. To overcome this problem, nanoparticle delivery system is very important to improve water solubility.

Nanoparticle delivery systems can be defined as drug-carrying particles having size in nanometers (nm). This system is more superior for reducing toxicity, improving solubility, and bioavailability of drugs. One of the most popular techniques for encapsulation of drug into NPs is the emulsion solvent evaporation technique. Previous studies had applied this strategy for natural products encapsulation such as juglone [43], caffeic acid phenethyl ester (CAPE) [21], and curcumin [44]. In this study, EEP-NPs were formulated and both their physiochemical and biological properties characterized. The ratio of EEP and PLGA was investigated in terms of mean particle size, particle surface charge, polydispersity index (PDI), surface morphology, encapsulation efficiency (EE), loading capacity (LC), and releasing profiles. All formulations of NPs demonstrated particles in nanometer scale according to elsewhere [18]. The zeta potential of NPs from all formulations was negative charge. These results implied the presence of ionized carboxyl groups of PLGA on the surface of NPs. Likewise, the zeta potential value of PLGA dissolved in neutral buffer without any PVA was −45 mV. However, the zeta potential value became less negative or close-to-neutral charge when PVA surrounded the PLGA nanoparticles [45]. Nanoparticles with zeta potential values greater than +30 mV or less than −30 mV typically have high degrees of stability [46]. Previous study used the chitosan-coated PLGA nanoparticles and improved the zeta potential from −2.8 mV to +33.47 mV [46]. The PDI values of EEP-NP 1 and EEP-NP 2 were nearly monodispersed, PDI values range from 0.1 to 0.7. On the contrary, the PDI value of EEP-NP 3 indicated a broad range of particle size and non-monomodal distribution, PDI value more than 0.7 [47]. Lyophilized form of NPs were kept at −20°C and dissolved immediately prior to use due to the prevention of NPs aggregation.

The high EE may be related to the EEP miscibility in PLGA. Concordance to previous study, the high miscibility of drugs and polymer interaction leads to high drug encapsulation efficacy in nanoparticles [48]. Moreover, Budhian et al. reported that the increase in initial haloperidol to polymer ratio in the emulsion leads to an increase in the amount of free drug, while the amount of encapsulated drug remains constant [49]. The efficient encapsulation of small hydrophilic/amphiphilic molecules from natural products into PLGA microspheres using conventional emulsification methods has been reported [50].

EEP-NPs showed a typical two-phase release pattern. The initial burst release of EEP from EEP-NPs was found in the first 6 h of incubation period. This rapid phase of EEP releasing from NPs might be EEP absorbed on the surface of EEP-NPs [51]. The second phase of release was observed from 7 to 24 h. A sustained release of EEP was caused by EEP diffusion and polymer chain cleavage. The increasing burst release of EEP-NPs resulted from the reduction of PLGA/EEP ratio. Therefore, the lowest of PLGA/EEP ratio indicated that PLGA was not enough to encapsulate EEP. Most EEP was absorbed or close to the surface of EEP-NPs, which resulted in high burst release [52].

The cytotoxicity of EEP-NPs demonstrated that the EEP-NPs represented less toxicity to Vero cells. In line with several studies, EEP had no cytotoxic effect on Vero cells. For example, Brazilian green propolis displayed antiproliferative effect on lung cancer cell lines (A549), but it did not suppress the growth of Vero cells [53]. In addition, propolis collected from different geographic regions of Indonesia showed cytotoxicity against tumor cell lines (T47D, MCF-7, and HeLa) and did not inhibit the growth of Vero cells [54]. Moreover, many studies demonstrated that the encapsulation of drug into PLGA nanoparticles can decrease the toxicity of drug. Gómez-Sequeda et al. showed that the encapsulation of fluconazole into PLGA nanoparticles showed less toxicity to Vero cells when compare with its free form [55].

This study compared the antifungal activity between EEP in free form and EEP-NPs by MTT assay. This assay is based on the ability of a cell to convert MTT to formazan, it reflects metabolic cell activity rather than direct cell viability. EEP-NP 2 showed the greater inhibition effect on metabolic activity of *C. albicans* when compared with EEP in free form. This might have been occurred in the high solubility of EEP-NPs and exhibited the potential inhibitory effect on mitochondrial enzyme activity [56, 57]. Another relevant study by Gómez-Sequeda et al. demonstrated that the fluconazole-loaded PLGA nanoparticles represented higher antifungal activity than free fluconazole [55]. The number of viable proliferative yeast was evaluated by the CFU assay. At all concentrations of EEP in free form and EEP-NPs, there was no effect on the growth of *C. albicans.* Notably, we found the small colonies of EEP-NP-treated yeasts compared with the control group grown on the SDA plate after incubation for 48 h. This phenomenon can be explained by the trailing effect (TE) as a reduced but persistent yeast growth [58]. The mechanisms underlying TE have been described, e.g., inoculum size [59], the incubation temperature [60], genetic diversity in *C. albicans* [61], and upregulation of drug-resistant genes [62]. According to Mello et al., the Brazilian green propolis affected the cell wall of *C. albicans* and may be the main reason for the decrease in the rate of yeast budding [63]. Likewise, the decrease in the ergosterol content of *C. albicans* indicates that these organisms show trailing growth in the presence of *Cassia fistula* extracts [64]. Thus, a compensatory increase in cell wall chitin synthesis might be the other reason of TE.

Adhesion ability of *C. albicans* is an important first step for pathogenesis. It has been reported that the possible mode of action of phenolic compound was an anti-adhesion ability of *C. albicans*. Feldman et al. reported the A-type cranberry proanthocyanidins (AC-PACs) reduced *C. albicans* adherence to oral epithelial cells. The mode of action of AC-PACs involves the reduction of cell surface hydrophobicity of *C. albicans*. The hydrophobic interaction generally plays an important role for the adherence of *C. albicans* to host cells or abiotic surface; it is usually considered as a good predictor for adhesion ability [24]. Furthermore, other phenolic compounds were studied, e.g., caffeic acid derivatives which may inhibit 1,3-*β*-glucan synthase [65], and curcumin can disrupt the cell membrane of *C. albicans* [66]. Moreover, eugenol and methyleugenol showed a potential to inhibit ergosterol biosynthesis in *Candida* and subsequently damaging the cell membrane [67].

The deep investigation of the adhesion hyphal-related gene expression, *ALS3* and *HWP1*, was determined by real-time RT-PCR. We found that the reduction of *HWP1* and *ALS3* mRNA expression supported our hypothesis by resulting in the reduction of the adhesion ability, hyphal germination, and biofilm formation. Similarly to de Groot et al., *HWP1* and *ALS3* genes were important for adhesion and biofilm formation of *C. albicans* [68]. In a previous study, Shahzad et al. reported two polyphenol compounds, curcumin and pyrocatechol, which downregulated *HWP1* and *ALS3* expressions, leading to the decrease of the biofilm formation of *C. albicans* [69]. EEP-NP 2 was capable to reduce the *C. albicans* hyphal germination and consequently decrease the expression of *HWP1*. Simultaneously, the inhibitory effect and the invasive ability of *C. albicans* are reduced by the release of LDH from Vero cells, which is an indicator for the cell damage. It was clearly seen that the reduction of adhesion and hyphal formation by EEP-NP 2 decreased the ability of *C. albicans* to penetrate the Vero cell membrane. A study by Saville et al., described that the inhibiting hyphal germination in the early stage of infection significantly increased the survival of the host cells [70].

## 5. Conclusion

Ethanolic extract of propolis-loaded PLGA nanoparticles (EEP-NPs) were successfully synthesized by using the single emulsion solvent evaporation method. EEP-NP 2 possessed unique physicochemical properties and less cytotoxicity due to their biodegradable and biocompatible properties. Moreover, EEP-NP 2 exhibited inhibitory effects on the growth of *C. albicans*. Importantly, it altered the expression of gene-encoding virulence-associated hyphal adhesion proteins of *C. albicans*, causing the yeast to lose their characteristic features that enable colonization, germination, biofilm formation, and invasion. Finally, the ultimate challenge will be in developing the appropriate pharmaceutical drug and studying the animal models that are predictive in treating candidiasis in the future.

## Figures and Tables

**Table 1:** Physicochemical properties of EEP-NPs and polymer-NPs.

Formulations	Mean particle size (nm)	Zeta potential (mV)	PDI	Encapsulation efficiency (%)	Loading capacity (%)
EEP-NP 1	379.2 ± 21.6	−2.6 ± 1.4	0.36 ± 0.02	92.1 ± 0.5	28.6 ± 1.1
Polymer-NP 1	424.7 ± 7.7	−1.4 ± 1.6	0.42 ± 0.01	—	—
EEP-NP 2	418.4 ± 35.3	−3.9 ± 0.5	0.57 ± 0.08	91.5 ± 0.9	42.0 ± 1.8
Polymer-NP 2	474.5 ± 47.7	−2.1 ± 1.0	0.54 ± 0.09	—	—
EEP-NP 3	229.5 ± 38.4	−1.2 ± 1.1	0.73 ± 0.13	89.90 ± 0.8	56.7 ± 3.4
Polymer-NP 3	178.2 ± 88.9	−2.2 ± 1.1	0.96 ± 0.04	—	—

## Data Availability

The data used to support the findings of this study are available from the corresponding author upon request.

## Supplementary Materials

Supplementary Materials Supplementary Table 1: parameters for EEP-NPs and polymer-NPs preparation. Supplementary Table 2: list and sequences of primers [71, 72]. Supplementary Figure 1: EEP-NP 2-inhibited *C. albicans* hyphal germination. Supplementary Figure 2: EEP-NP 2-induced cell death in *C. albicans*.
