# Supplementary material for: In Vitro Antifungal and Antivirulence Activities of Biologically Synthesized Ethanolic Extract of Propolis-Loaded PLGA Nanoparticles against Candida albicans
Source: Evid Based Complement Alternat Med. 2019 Nov 30;2019:3715481. doi: 10.1155/2019/3715481 (PMC6907039; doi:10.1155/2019/3715481)
Supplement: Supplementary Materials — Supplementary Table 1: parameters for EEP-NPs and polymer-NPs preparation. Supplementary Table 2: list and sequences of primers [71, 72]. Supplementary Figure 1: EEP-NP 2-inhibited C. albicans hyphal germination. Supplementary Figure 2: EEP-NP 2-induced cell death in C. albicans. [file 3715481.f1.zip › Final-supplementary data_Tragoolpua K November 14, 2019_ECAM_2932099.docx]

***In vitro* antifungal and antivirulence activities of biologically synthesized ethanolic extract of propolis-loaded PLGA nanoparticles against *Candida albicans***

Anupon Iadnut^1,2^, Ketsaya Mamoon ^1,2^, Patcharin Thammasit^1,2^, Sudjai Pawichai^1^, Singkome Tima^3^, Kanya Preechasuth^1,5^, Thida Kaewkod^4^, Yingmanee Tragoolpua^4^ and Khajornsak Tragoolpua^1,5^

**Supplementary Materials**

1. **Preparation of nanoparticles**

The composition of unloaded NPs is summarized in Supplementary Table 1. Three formulations were investigated by the concentration of Poly (lactic-co-glycolic acid) (PLGA) and Ethanolic extract of propolis (EEP). The optimal condition in term of physicochemical properties of nanoparticles (NPs) was carried out as described in Table 1.

**Supplementary Table 1:** Parameters for EEP-NPs and Polymer-NPs preparation.

| Formulation | Ratio of  PLGA:EEP | Compositions | | | | |
| --- | --- | --- | --- | --- | --- | --- |
|  |  | PLGA (mg) | DCM (ml) | EEP (mg) | EtOH (ml) | 2% PVA (ml) |
| EEP-NP 1 | 2:1 | 100 | 1.5 | 50 | 0.5 | 4 |
| Polymer NP 1 | - | 100 | 1.5 | - | 0.5 | 4 |
| EEP-NP 2 | 1:1 | 50 | 1.5 | 50 | 0.5 | 4 |
| Polymer-NP 2 | - | 50 | 1.5 | - | 0.5 | 4 |
| EEP-NP 3 | 1:2 | 25 | 1.5 | 50 | 0.5 | 4 |
| Polymer-NP 3 | - | 25 | 1.5 | - | 0.5 | 4 |

1. **Primer sequences used for SYBR-green RT-PCR**

The list and sequences of primers for hyphal-adhesion related gene expression of *Candida albicans* were shown in Supplementary Table 2.

**Supplementary Table 2:** List and sequences of primers

| Primer | Sequence | References |
| --- | --- | --- |
| *HWP1*_F  *HWP1*_R | 5’-TGGTGCTATTACTATTCCGG-3’  5’- CAATAATAGCAGCACCGAAG -3’ | [73]  [73] |
| *ALS3*_F  *ALS3*_R | 5’-GCAACGTGCACCTTTCACAT-3’  5’-TCGCGGTTAGGATCGAATGG-3’ |  |
| *ACT1*_F  *ACT1*_R | 5’-AGCTTTGTTCAGACCAGCTGATT-3’  5’- GGAGTTGAAAGTGGTTTGGTCAA -3’ | [74]  [74] |

1. **EEP-NP 2 reduced the hyphal germination of *C. albicans***

*C. albicans* were treated with various concentrations of EEP-NP 2 and incubated in inducing medium. We found that the hyphal extension was reduced in EEP-NP 2 treated-*C. albicans* by a dose-dependent manner after 4 h of incubation (Supplementary Figures 1a-e).


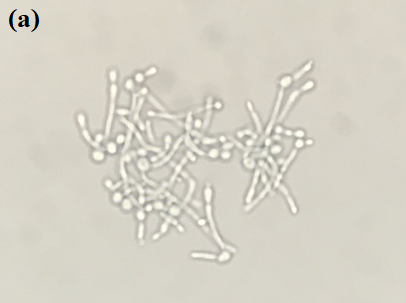
 **
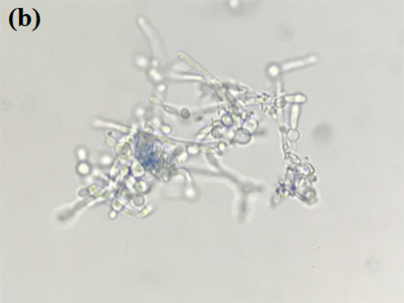
** **
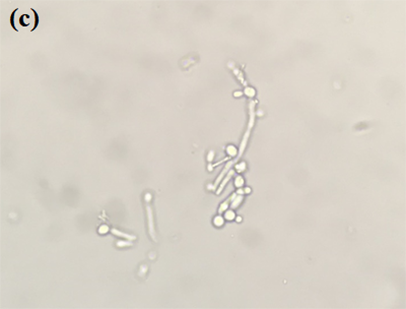
**


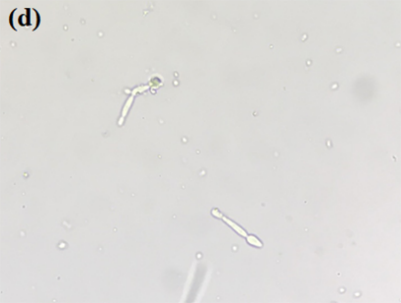

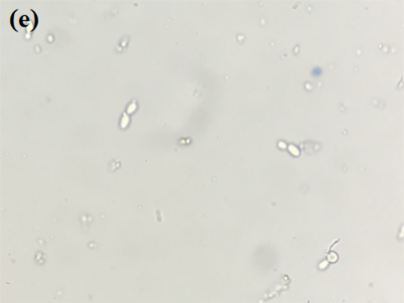


**Supplementary Figure 1:** EEP-NP2 inhibited *C. albicans* hyphal germination. Treated yeasts were incubated in 10% FBS-RPMI 1640 for 4 h. (a) untreated, (b) Polymer-NP 2, (c) 0.625 mg/ml, (d) 1.25 mg/ml, (e) 2.5 mg/ml of EEP-NP 2.

1. According to the reviewer’s suggestion, we have some preliminary data about cell death in yeasts. *C. albicans* were treated with EEP-NP2 for 24 h and stained with 4′,6-diamidino-2-phenylindole (DAPI) and propidium iodide (PI). DAPI is a fluorescent dye that binds strongly to adenine–thymine rich regions in DNA whereas PI is a membrane impermeable fluorescent dye. This dye stains dead cells by binding to DNA as a result of porous membrane. As shown in Supplementary Figure 2, EEP-NP2 affected the integrity of the cell membrane and resulted in more positive cells for PI staining when compare with untreated group (Fig. S2a) and polymer-NP2 control group (Fig. S2c). Treated-yeasts with 70% EtOH for 20 min was performed as a positive control group (Fig. S2b).


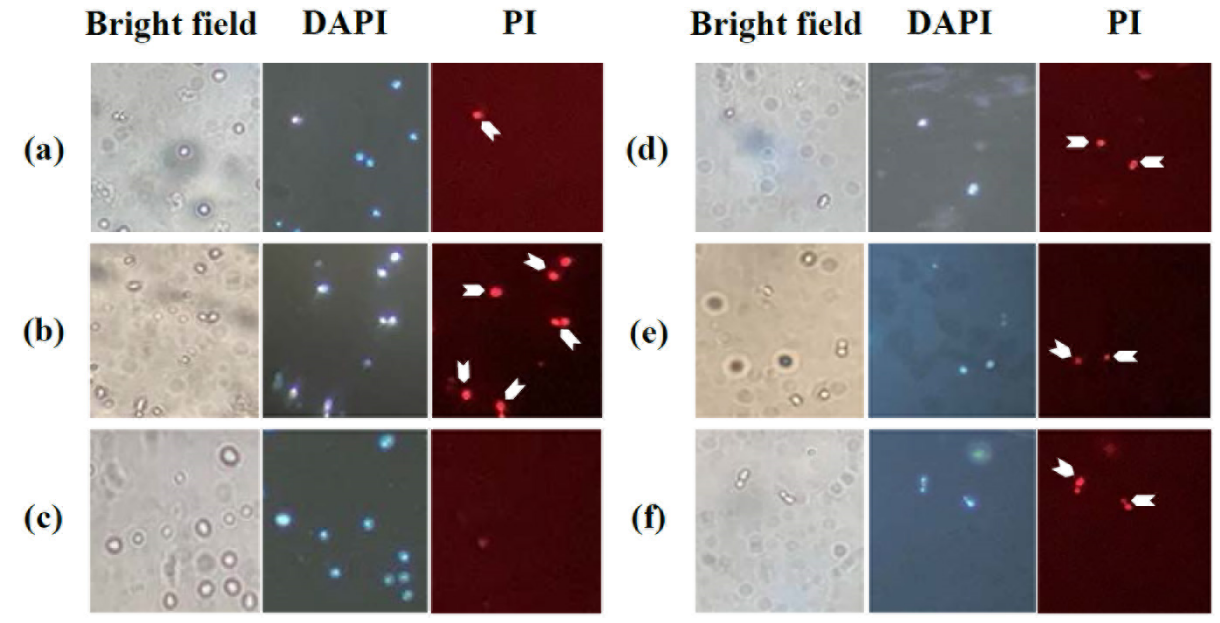


**Supplementary Figure 2** EEP-NP2 induced cell death in *C. albicans*. Yeasts were incubated with (a) RPMI media only (b) 70% Ethanol (c) polymer-NP 2 (d) 0.625 mg/ml of EEP-NP 2 (e) 1.25 mg/ml of EEP-NP 2 and (f) 2.5 mg/ml of EEP-NP 2. Then all condition were stained with DAPI and propidium iodide (PI). The yeast cells were observed under fluorescence microscope (Zeiss Axiolab, Germany) at 400 × magnification. White arrows represent PI positive cells.
