# Supplementary figures and images for: In Vitro Antifungal and Antivirulence Activities of Biologically Synthesized Ethanolic Extract of Propolis-Loaded PLGA Nanoparticles against Candida albicans
Source: Evid Based Complement Alternat Med. 2019 Nov 30;2019:3715481. doi: 10.1155/2019/3715481 (PMC6907039; doi:10.1155/2019/3715481)

**Supplementary Figure 1a**


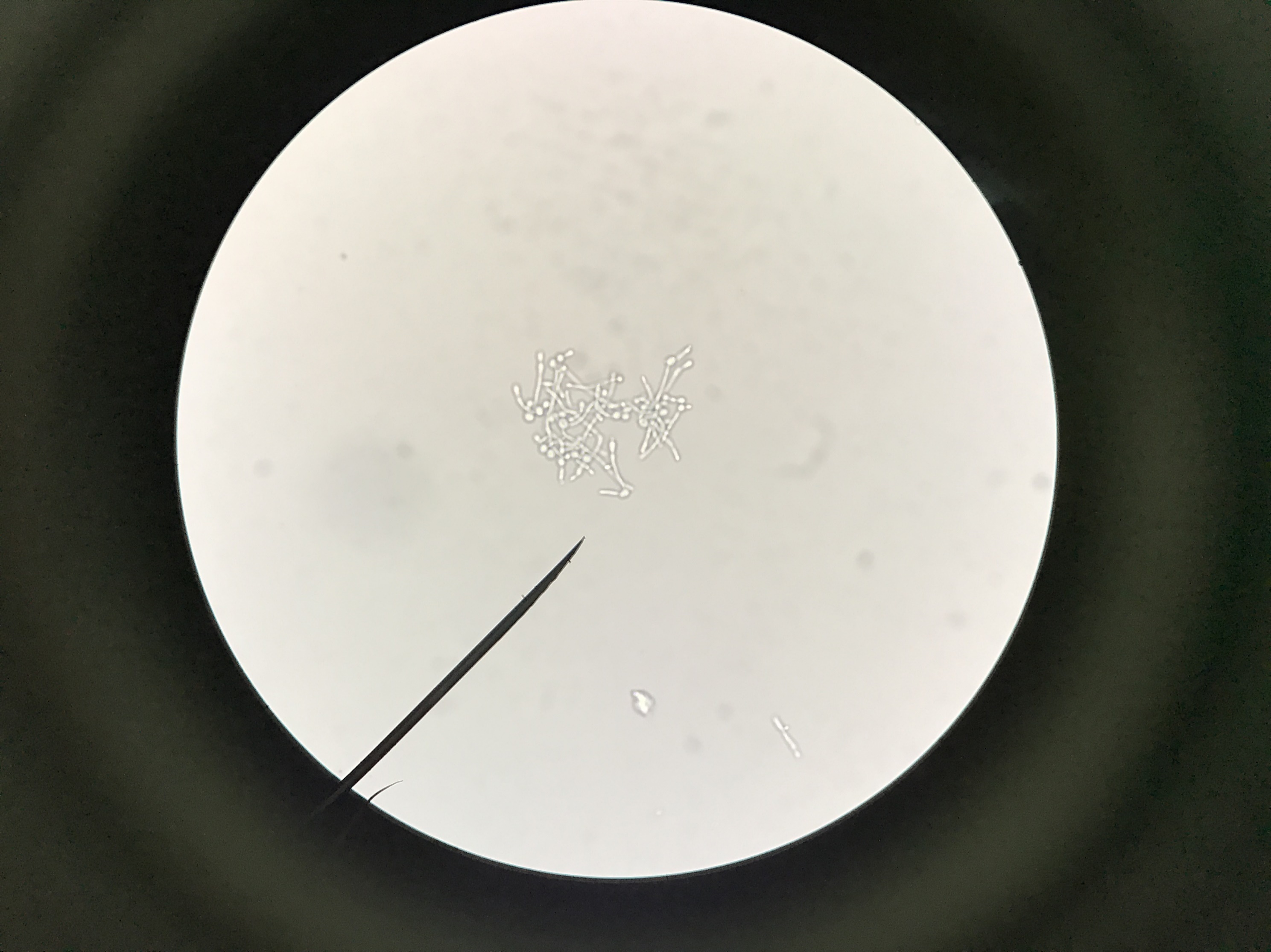


**(a)**

Supplement: Supplementary Materials — Supplementary Table 1: parameters for EEP-NPs and polymer-NPs preparation. Supplementary Table 2: list and sequences of primers [71, 72]. Supplementary Figure 1: EEP-NP 2-inhibited C. albicans hyphal germination. Supplementary Figure 2: EEP-NP 2-induced cell death in C. albicans. [file 3715481.f1.zip › Supplementary Figure 1a_November 16, 2019_ECAM_2932124.docx]

**Supplementary Figure 1b**


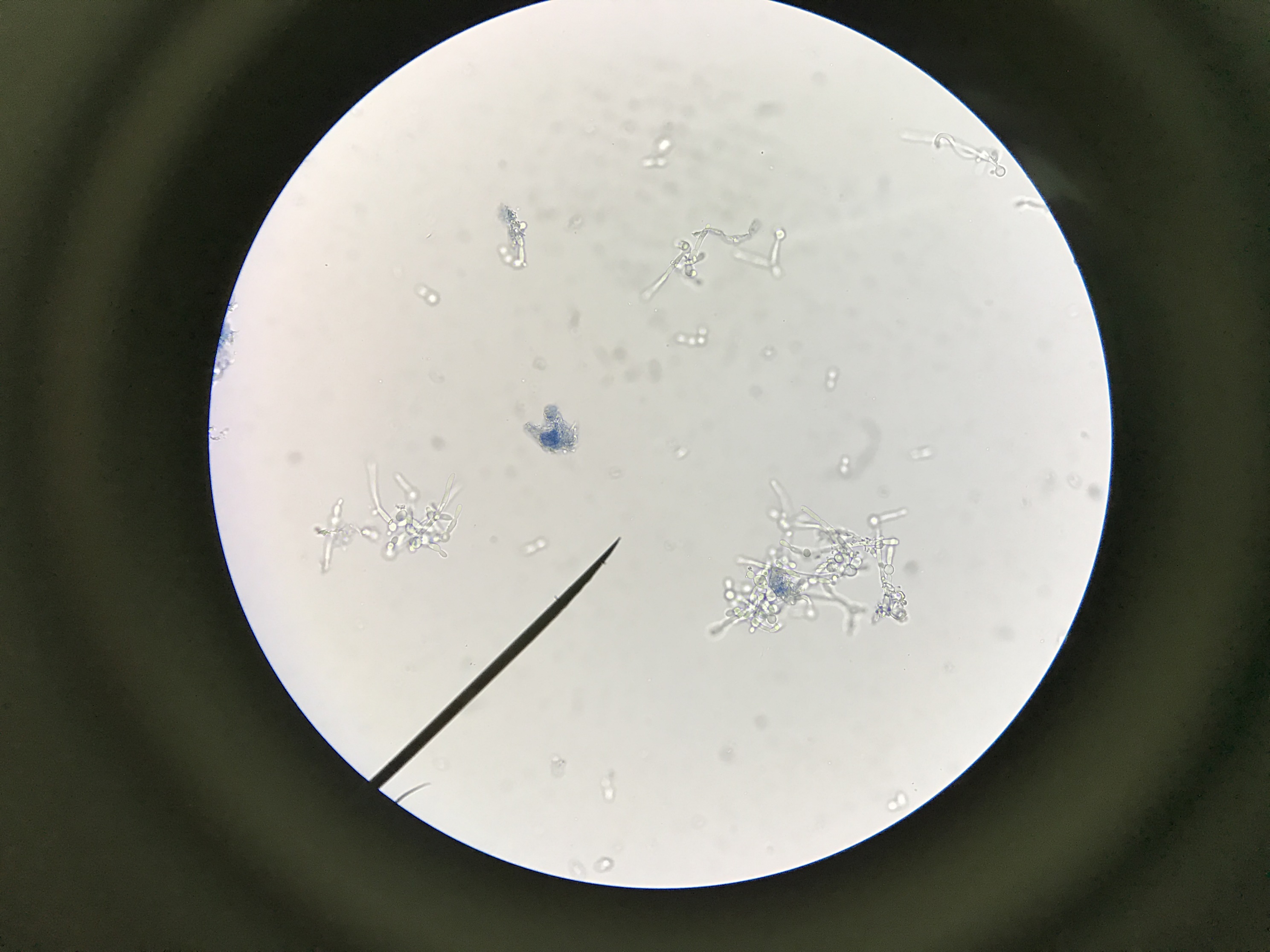


**(b)**

Supplement: Supplementary Materials — Supplementary Table 1: parameters for EEP-NPs and polymer-NPs preparation. Supplementary Table 2: list and sequences of primers [71, 72]. Supplementary Figure 1: EEP-NP 2-inhibited C. albicans hyphal germination. Supplementary Figure 2: EEP-NP 2-induced cell death in C. albicans. [file 3715481.f1.zip › Supplementary Figure 1b_November 16, 2019_ECAM_2932125.docx]

**Supplementary Figure 1c**


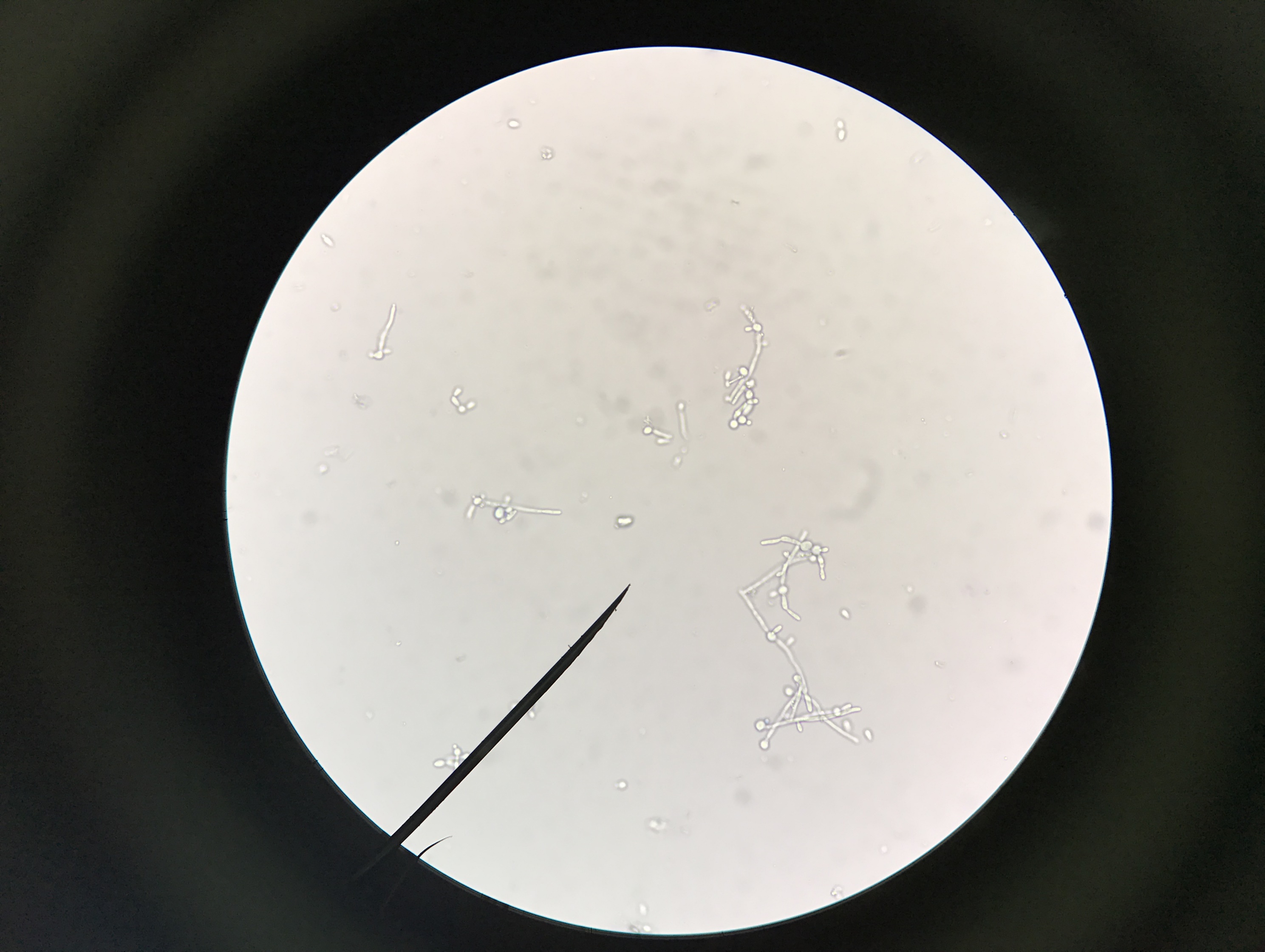


**(c)**

**(b)**

Supplement: Supplementary Materials — Supplementary Table 1: parameters for EEP-NPs and polymer-NPs preparation. Supplementary Table 2: list and sequences of primers [71, 72]. Supplementary Figure 1: EEP-NP 2-inhibited C. albicans hyphal germination. Supplementary Figure 2: EEP-NP 2-induced cell death in C. albicans. [file 3715481.f1.zip › Supplementary Figure 1c_November 16, 2019_ECAM_2932126.docx]

**Supplementary Figure 1d**


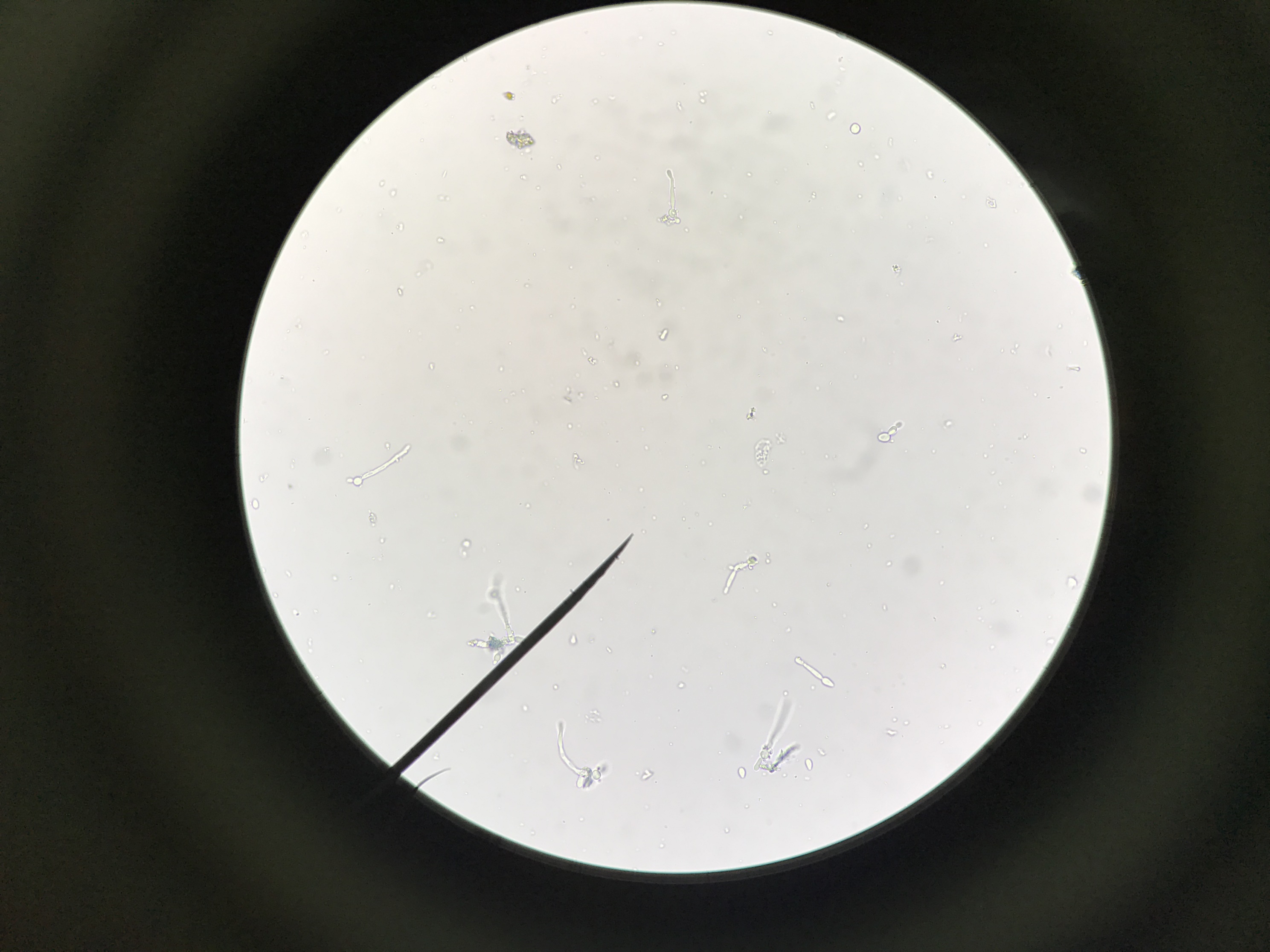


**(d)**

Supplement: Supplementary Materials — Supplementary Table 1: parameters for EEP-NPs and polymer-NPs preparation. Supplementary Table 2: list and sequences of primers [71, 72]. Supplementary Figure 1: EEP-NP 2-inhibited C. albicans hyphal germination. Supplementary Figure 2: EEP-NP 2-induced cell death in C. albicans. [file 3715481.f1.zip › Supplementary Figure 1d_November 16, 2019_ECAM_2932127.docx]

**Supplementary Figure 1e**


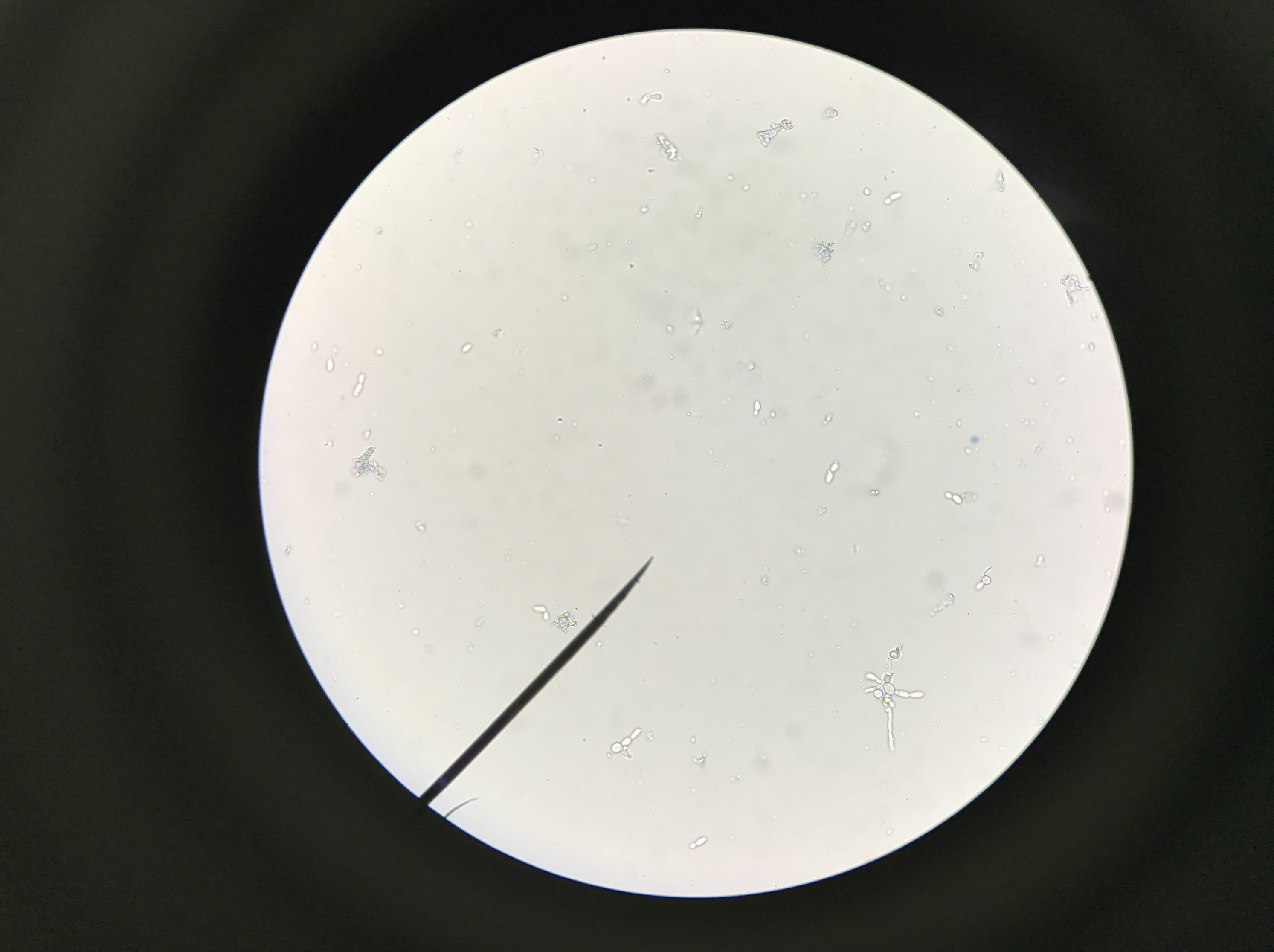


**(e)**

Supplement: Supplementary Materials — Supplementary Table 1: parameters for EEP-NPs and polymer-NPs preparation. Supplementary Table 2: list and sequences of primers [71, 72]. Supplementary Figure 1: EEP-NP 2-inhibited C. albicans hyphal germination. Supplementary Figure 2: EEP-NP 2-induced cell death in C. albicans. [file 3715481.f1.zip › Supplementary Figure 1e_November 16, 2019_ECAM_2932128.docx]

**Supplementary Figure 2a**

**
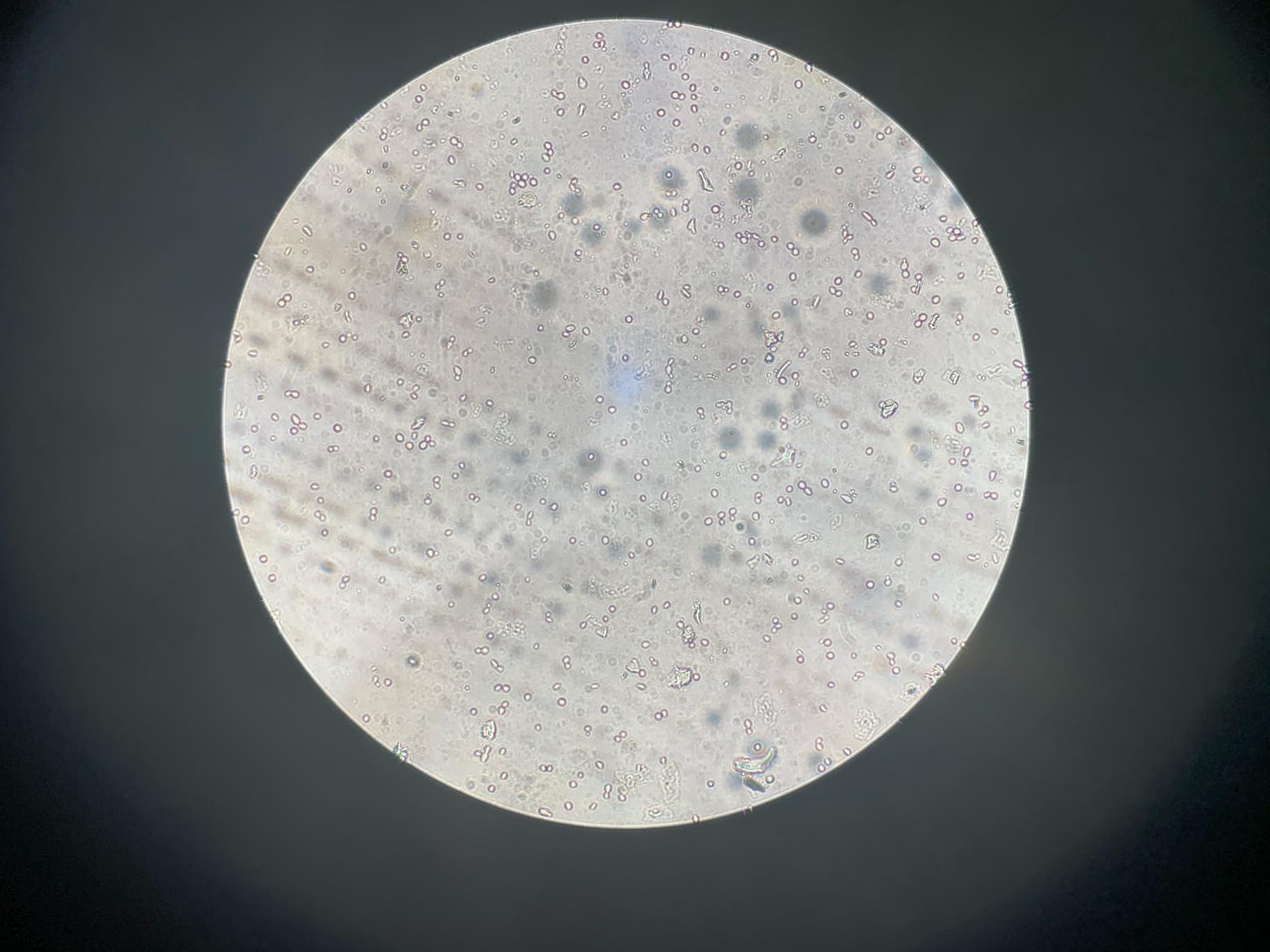

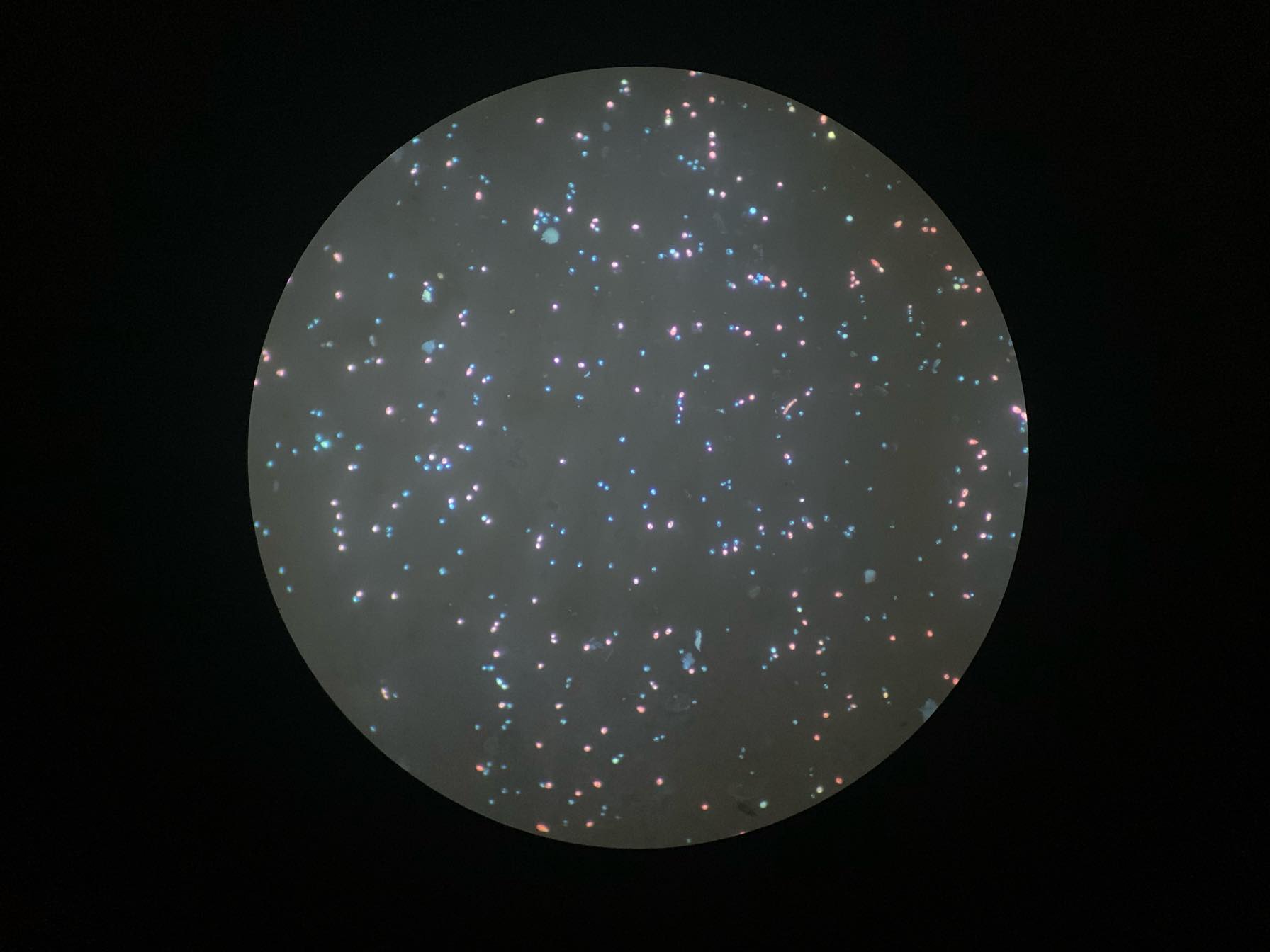

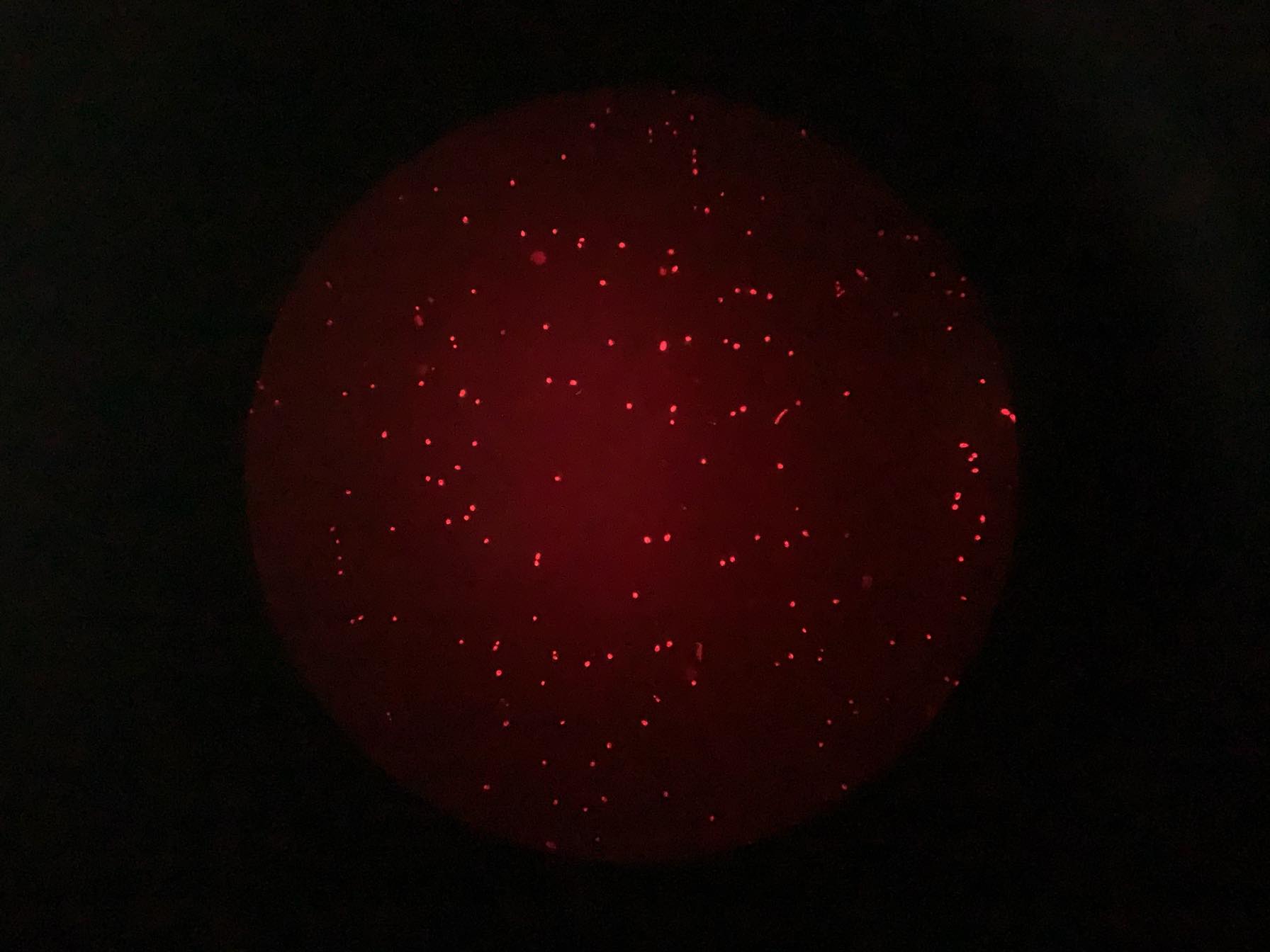
**

**Bright field**

**DAPI**

**PI**

Supplement: Supplementary Materials — Supplementary Table 1: parameters for EEP-NPs and polymer-NPs preparation. Supplementary Table 2: list and sequences of primers [71, 72]. Supplementary Figure 1: EEP-NP 2-inhibited C. albicans hyphal germination. Supplementary Figure 2: EEP-NP 2-induced cell death in C. albicans. [file 3715481.f1.zip › Supplementary Figure 2a_November 16, 2019_ECAM_2932129.docx]

**Supplementary Figure 2b**

**
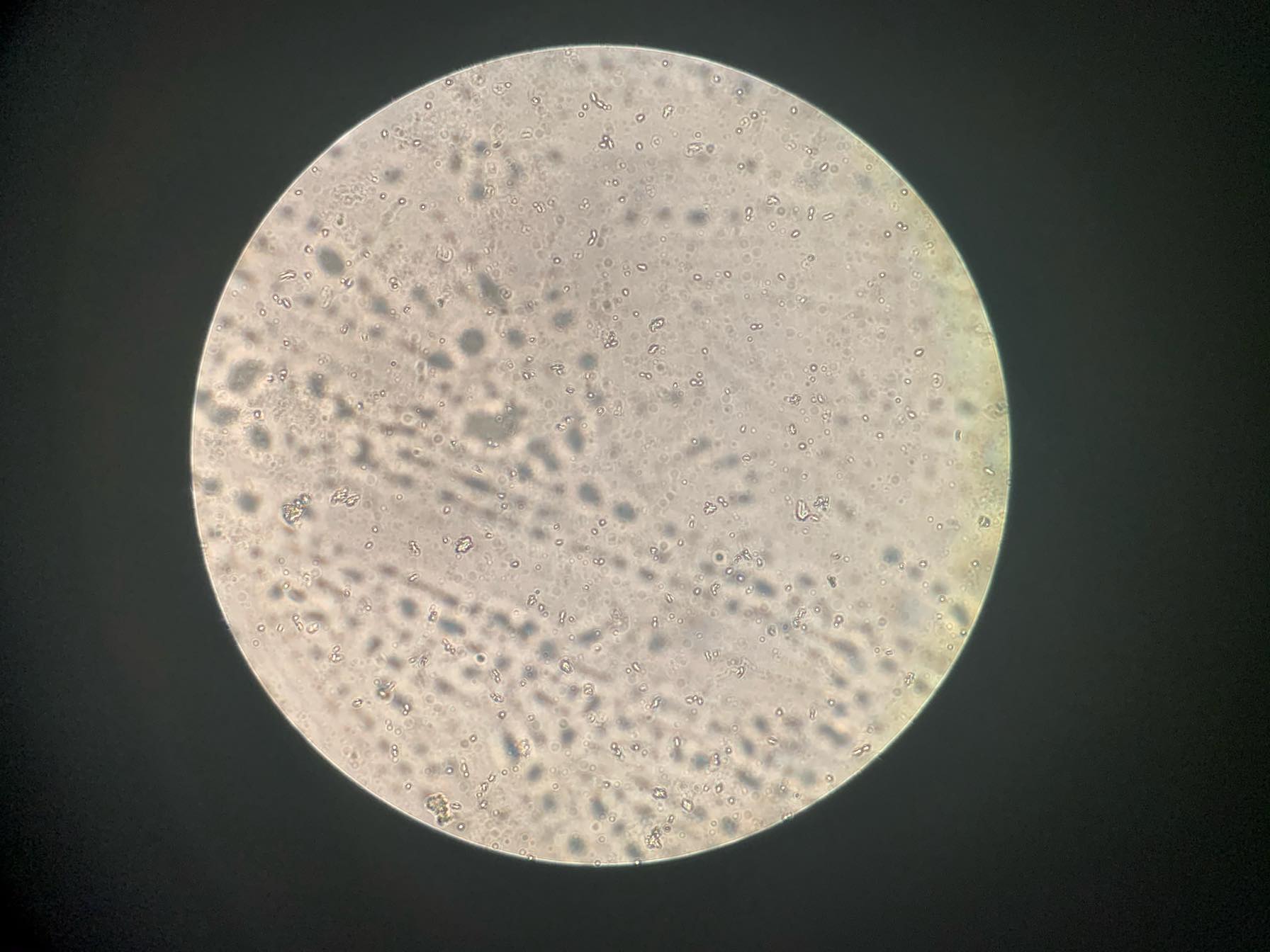

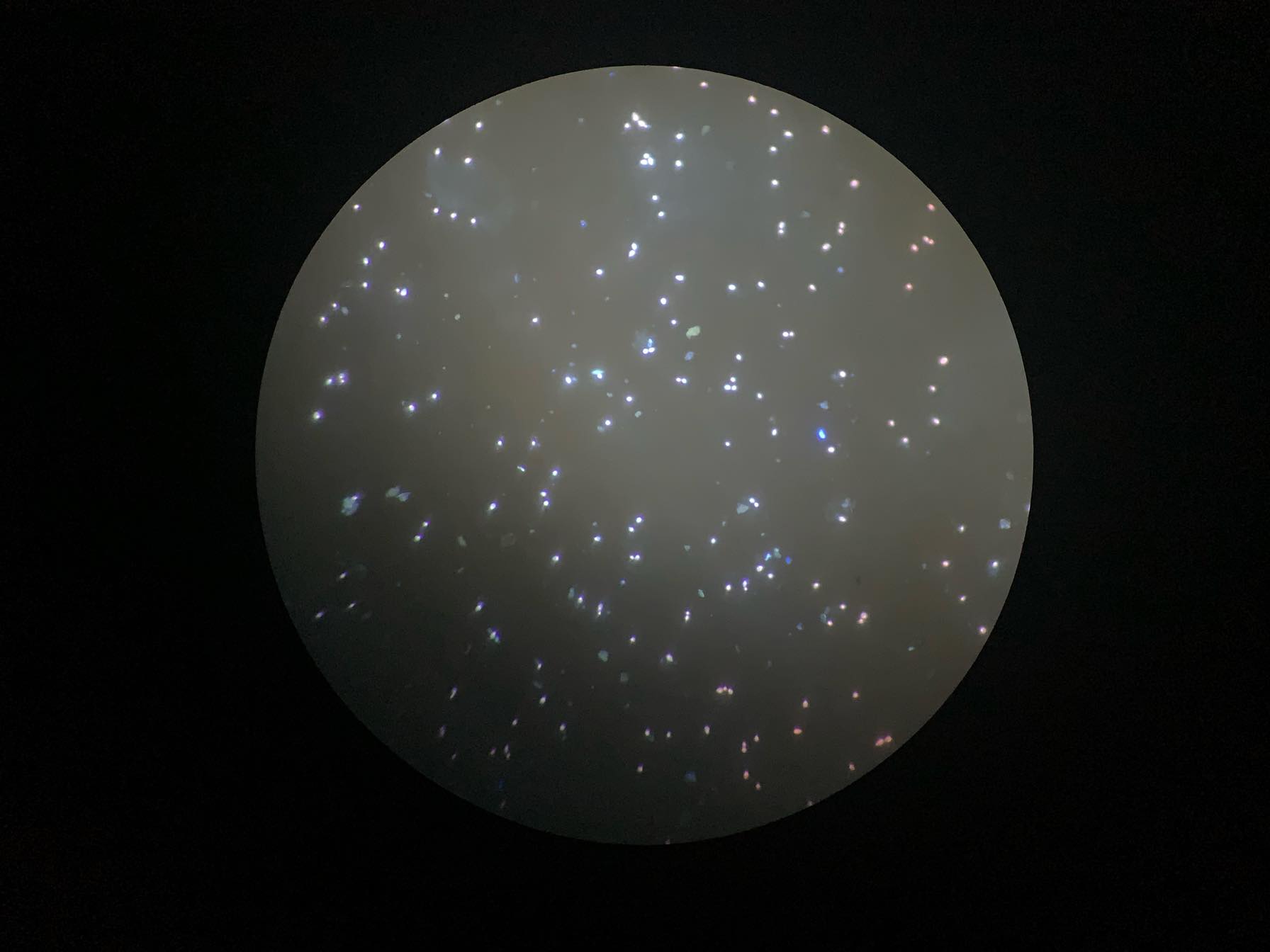

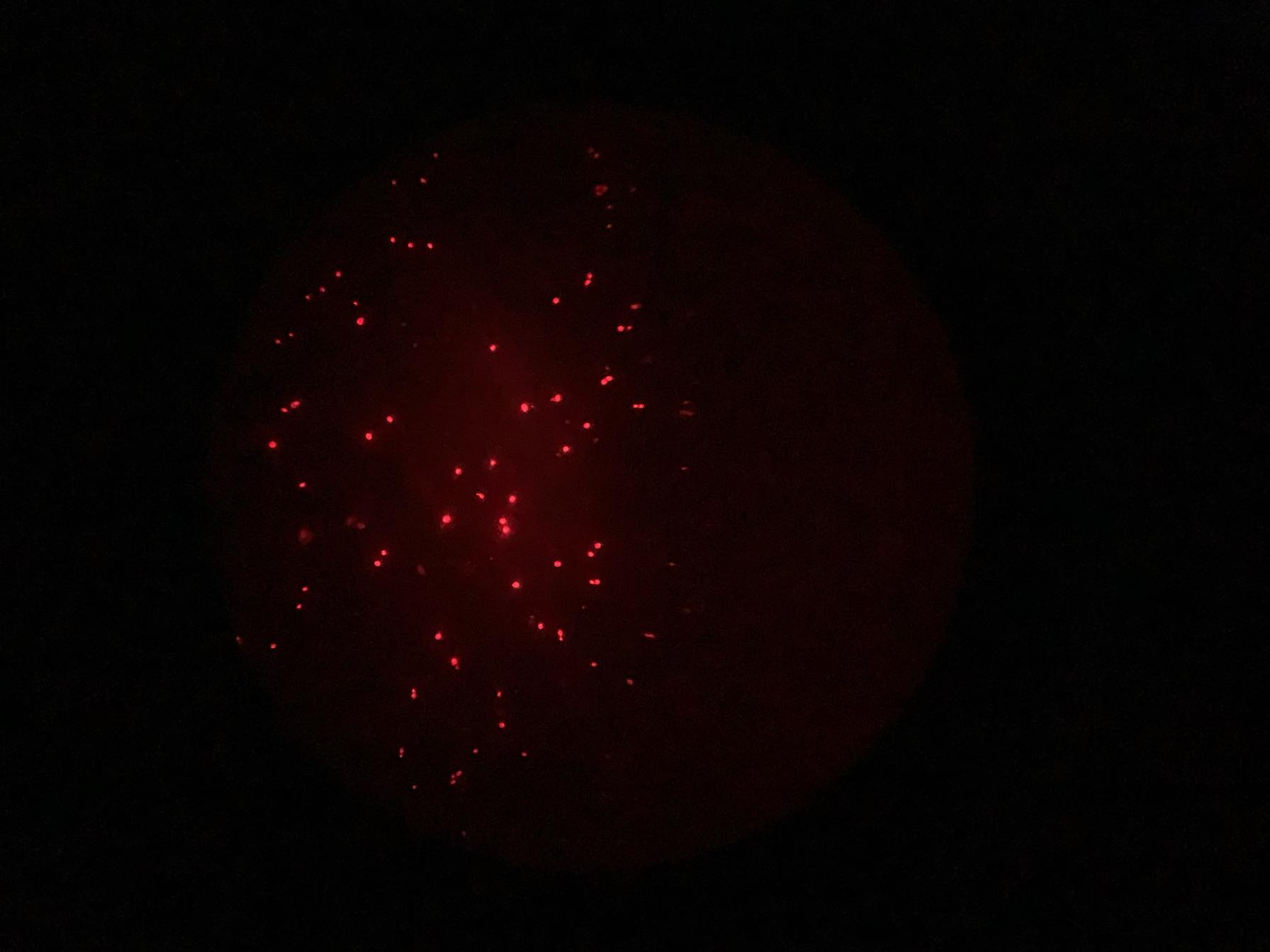
**

**Bright field**

**DAPI**

**PI**

Supplement: Supplementary Materials — Supplementary Table 1: parameters for EEP-NPs and polymer-NPs preparation. Supplementary Table 2: list and sequences of primers [71, 72]. Supplementary Figure 1: EEP-NP 2-inhibited C. albicans hyphal germination. Supplementary Figure 2: EEP-NP 2-induced cell death in C. albicans. [file 3715481.f1.zip › Supplementary Figure 2b_November 16, 2019_ECAM_2932130.docx]

**Supplementary Figure 2c**

**
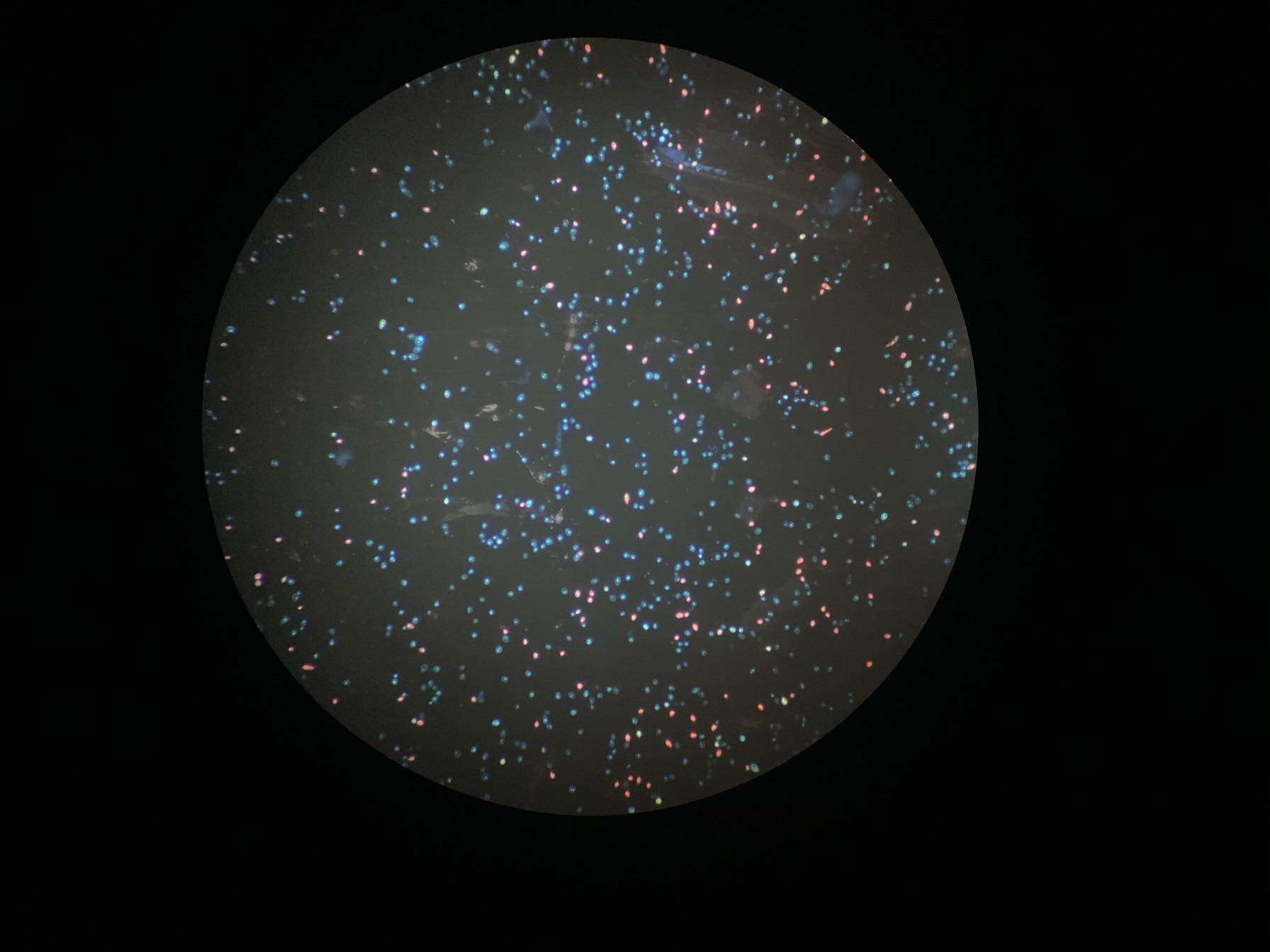

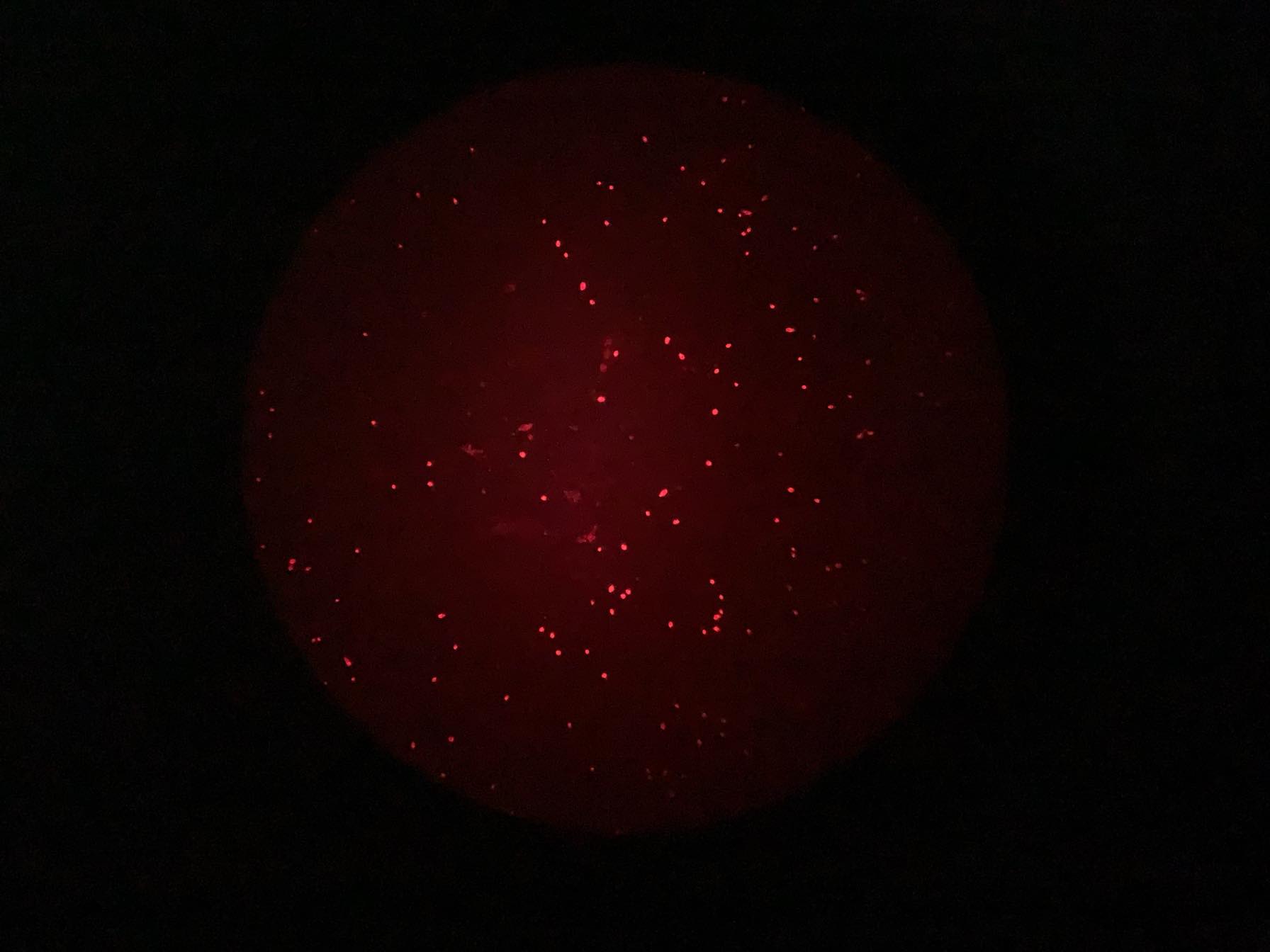

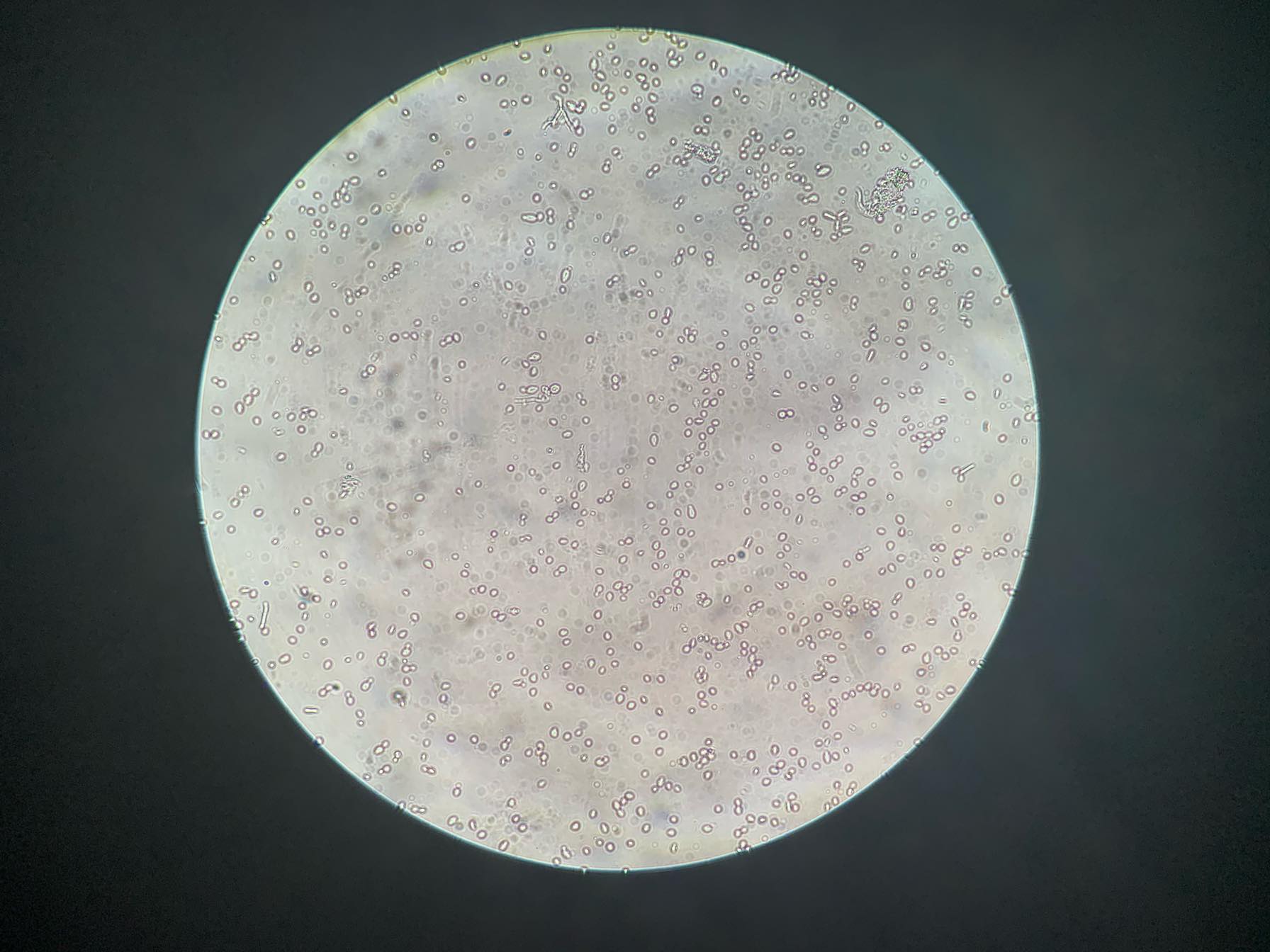
**

**Bright field**

**DAPI**

**PI**

Supplement: Supplementary Materials — Supplementary Table 1: parameters for EEP-NPs and polymer-NPs preparation. Supplementary Table 2: list and sequences of primers [71, 72]. Supplementary Figure 1: EEP-NP 2-inhibited C. albicans hyphal germination. Supplementary Figure 2: EEP-NP 2-induced cell death in C. albicans. [file 3715481.f1.zip › Supplementary Figure 2c_November 16, 2019_ECAM_2932131.docx]

**Supplementary Figure 2d**

**
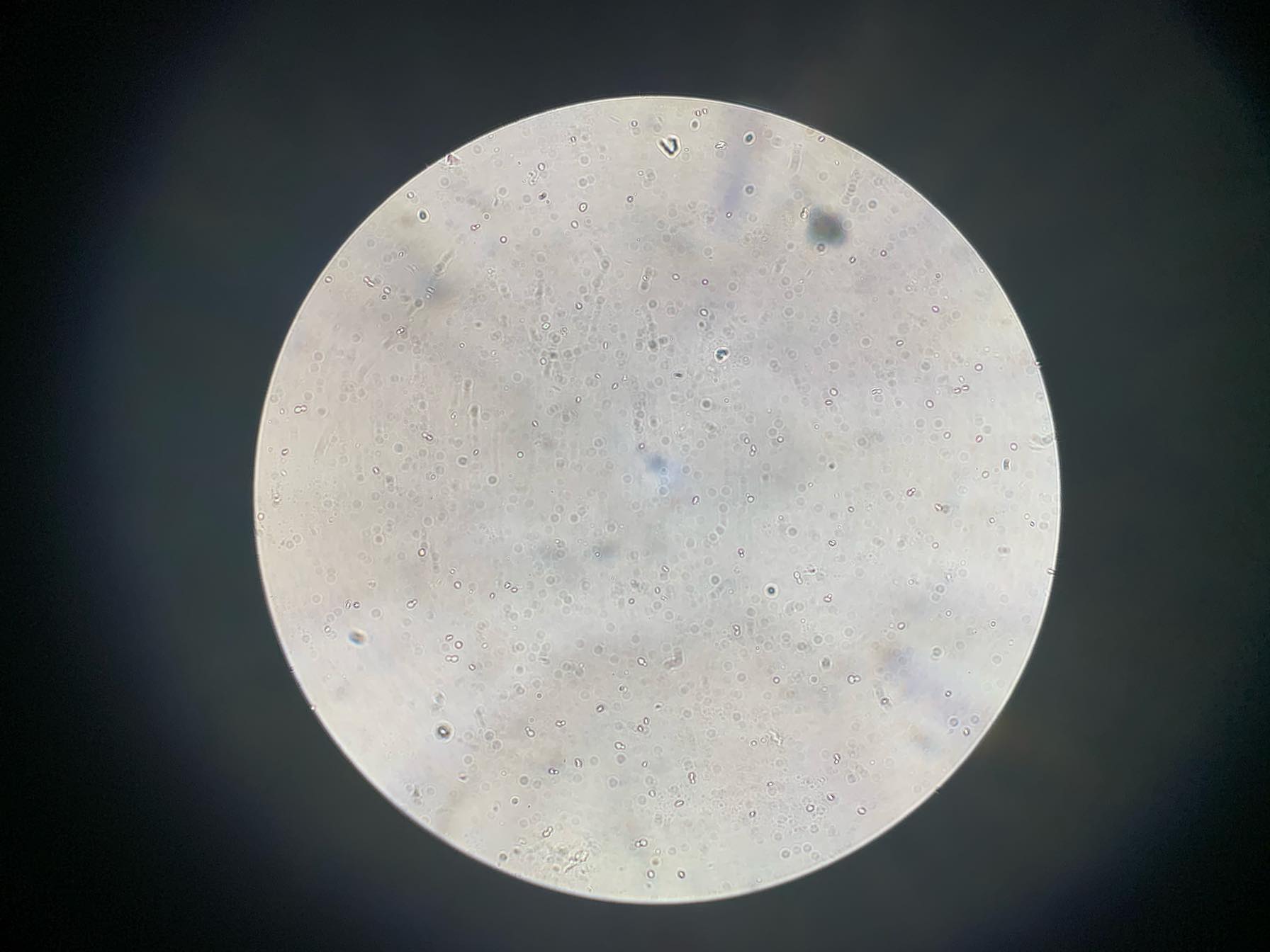

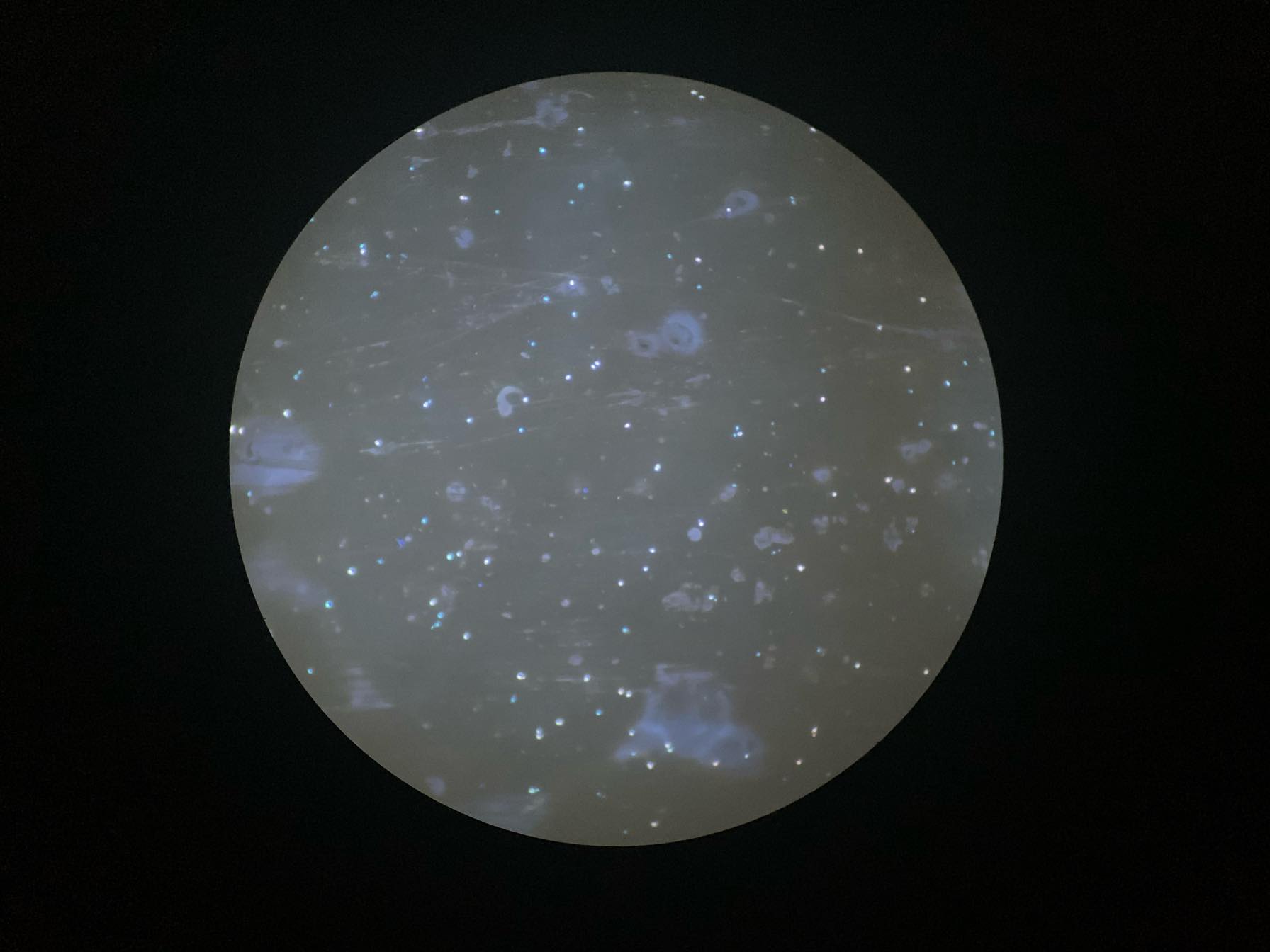

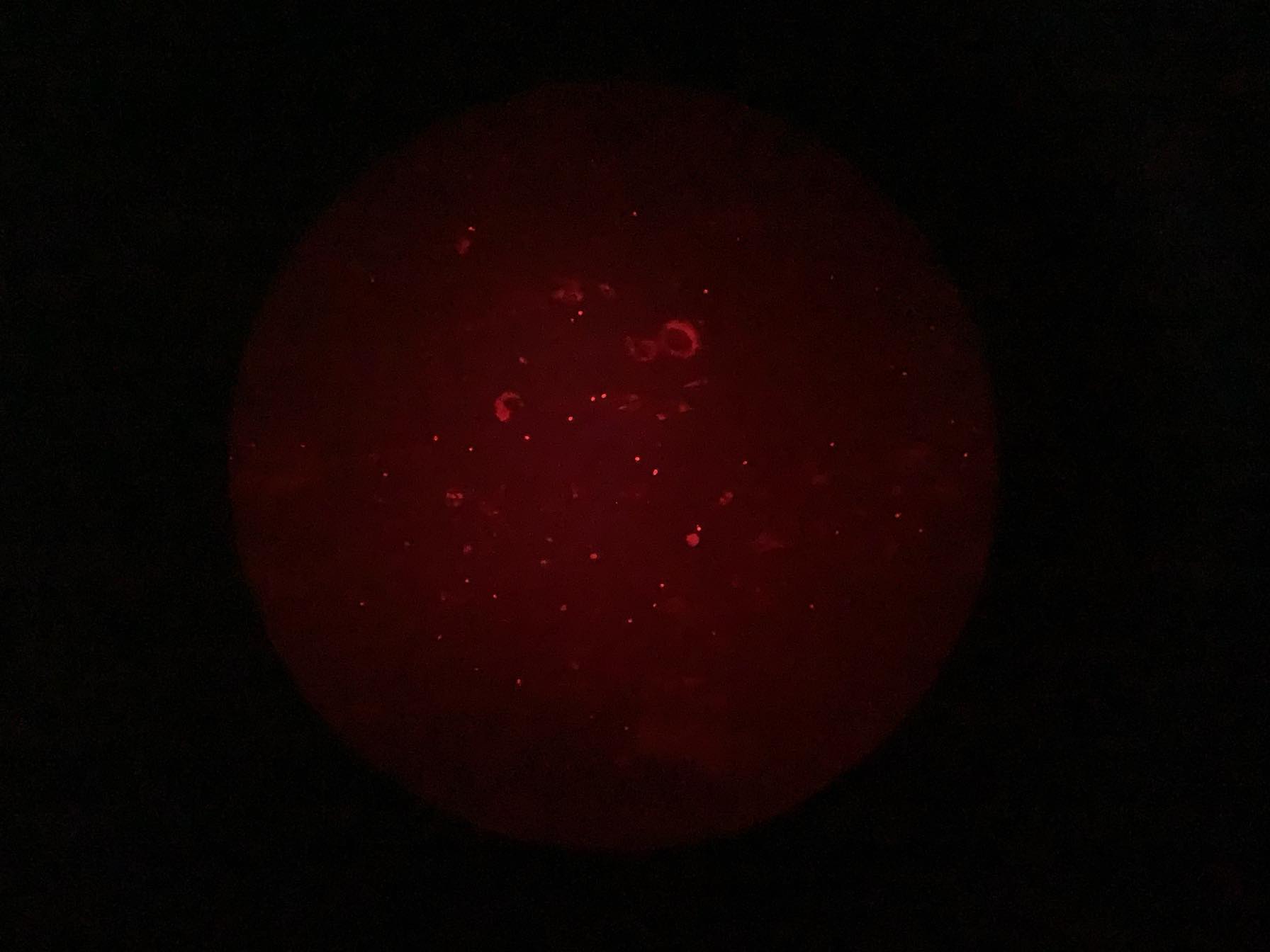
**

**Bright field**

**DAPI**

**PI**

Supplement: Supplementary Materials — Supplementary Table 1: parameters for EEP-NPs and polymer-NPs preparation. Supplementary Table 2: list and sequences of primers [71, 72]. Supplementary Figure 1: EEP-NP 2-inhibited C. albicans hyphal germination. Supplementary Figure 2: EEP-NP 2-induced cell death in C. albicans. [file 3715481.f1.zip › Supplementary Figure 2d_November 16, 2019_ECAM_2932132.docx]

**Supplementary Figure 2e**

**
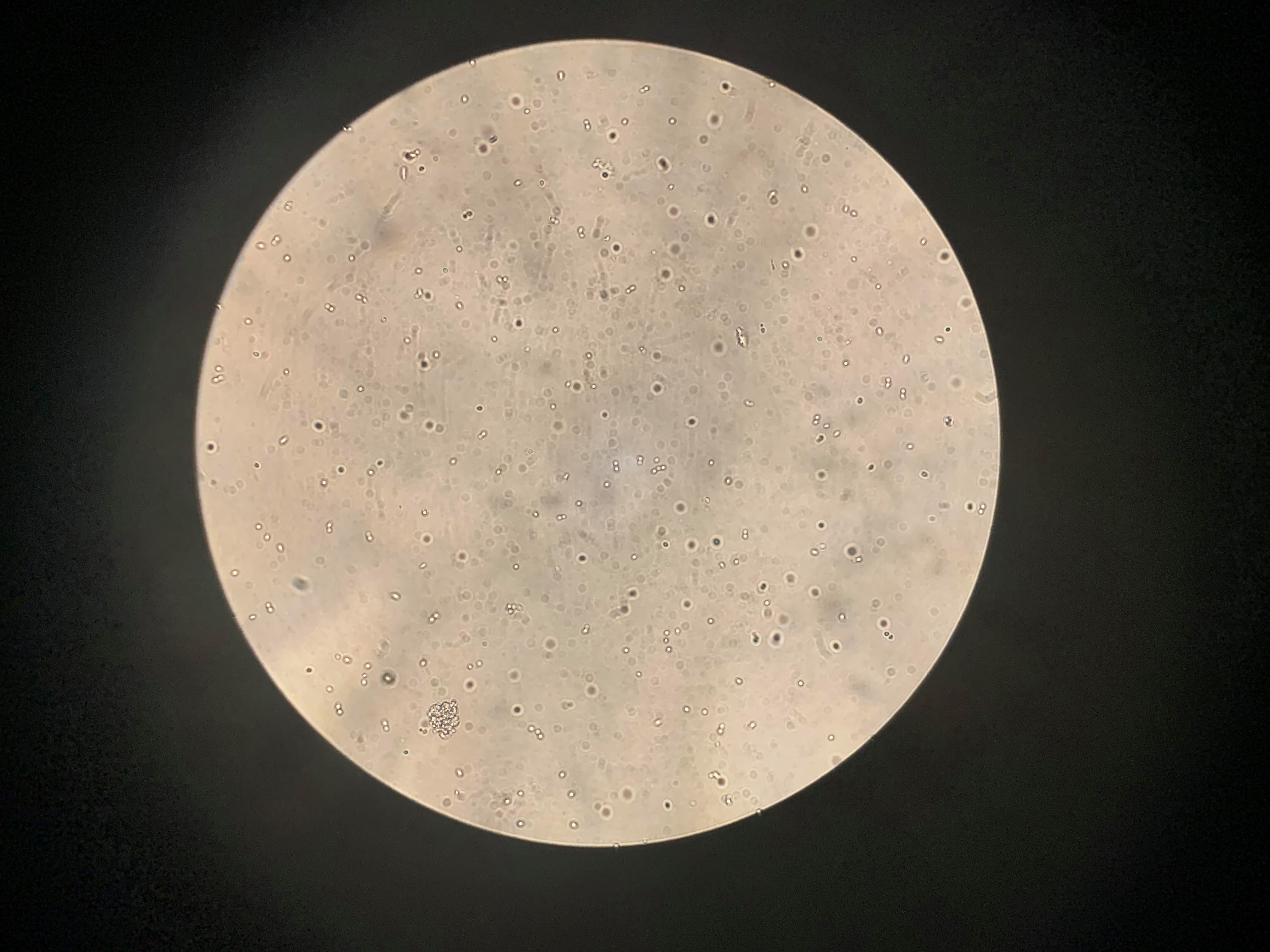

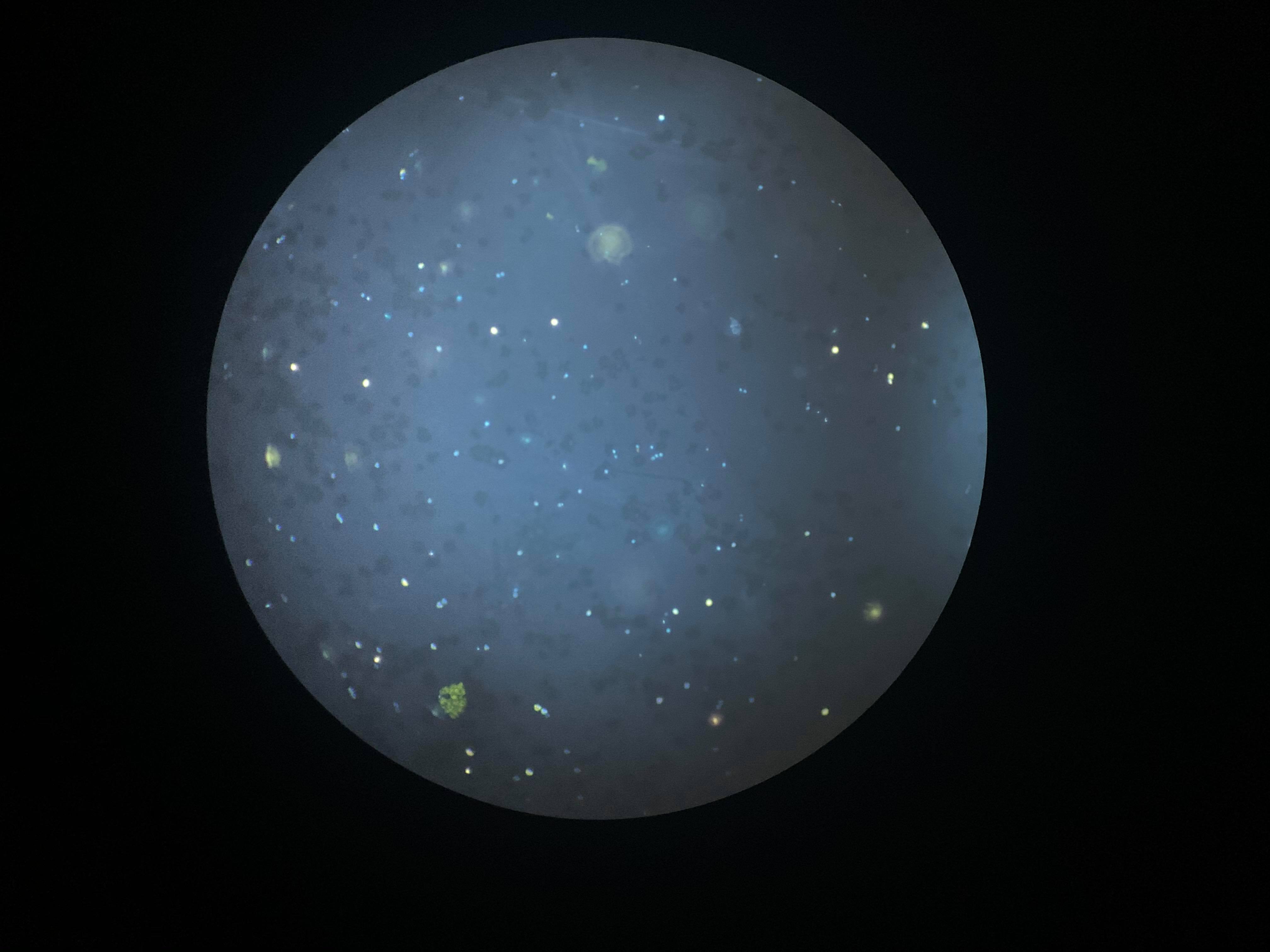

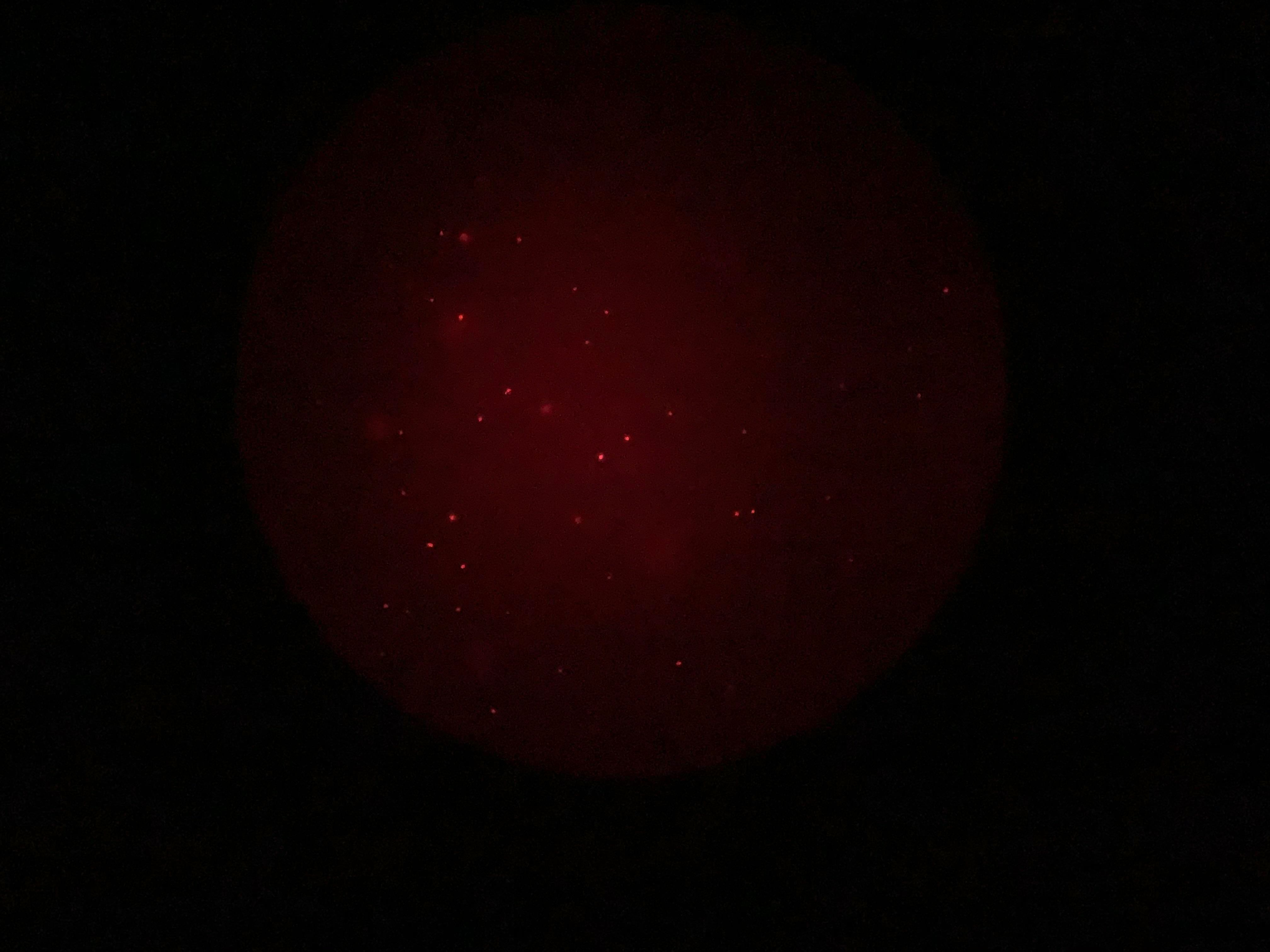
**

**Bright field**

**DAPI**

**PI**

Supplement: Supplementary Materials — Supplementary Table 1: parameters for EEP-NPs and polymer-NPs preparation. Supplementary Table 2: list and sequences of primers [71, 72]. Supplementary Figure 1: EEP-NP 2-inhibited C. albicans hyphal germination. Supplementary Figure 2: EEP-NP 2-induced cell death in C. albicans. [file 3715481.f1.zip › Supplementary Figure 2e_November 16, 2019_ECAM_2932133.docx]

**Supplementary Figure 2f**

**
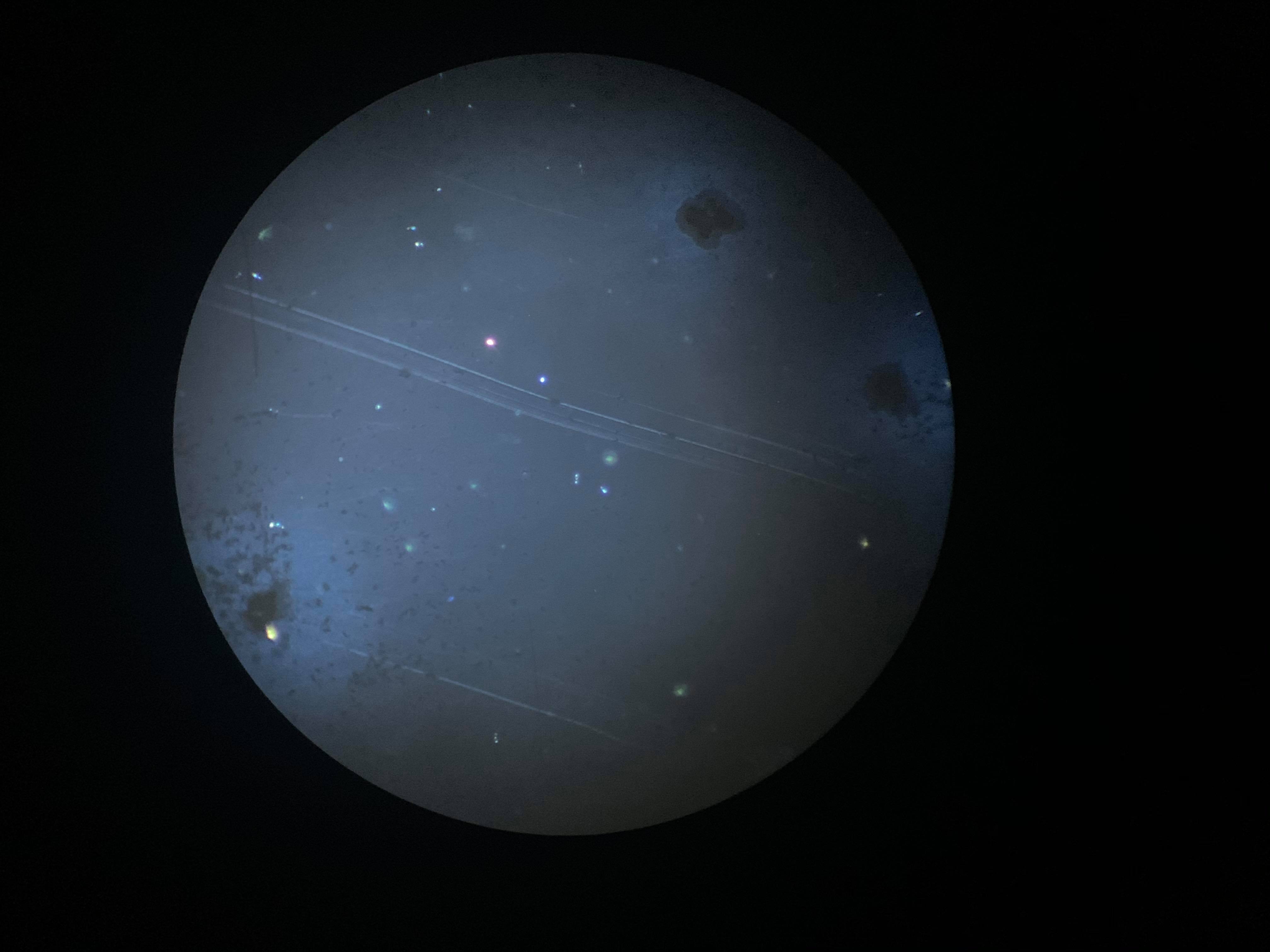

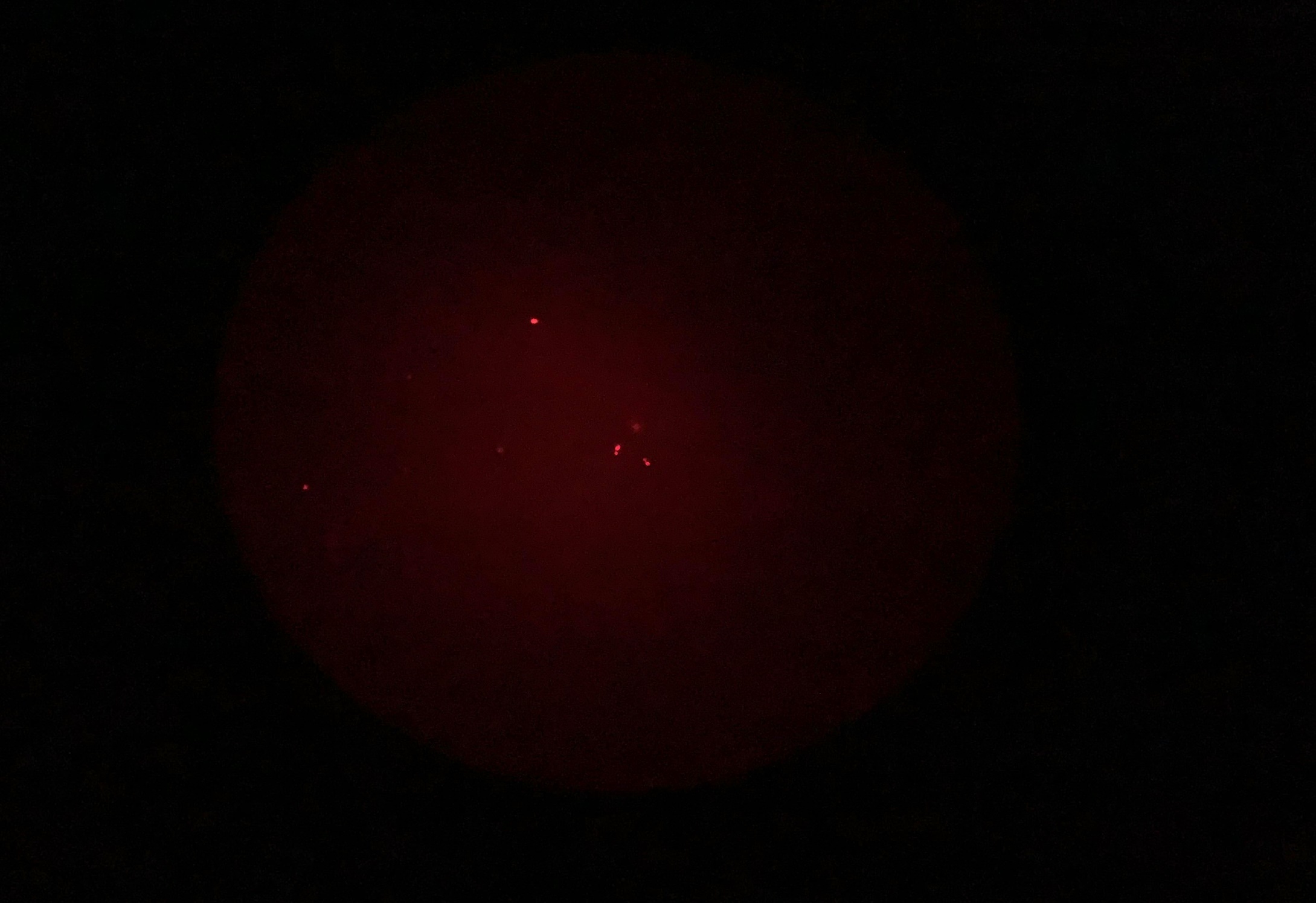

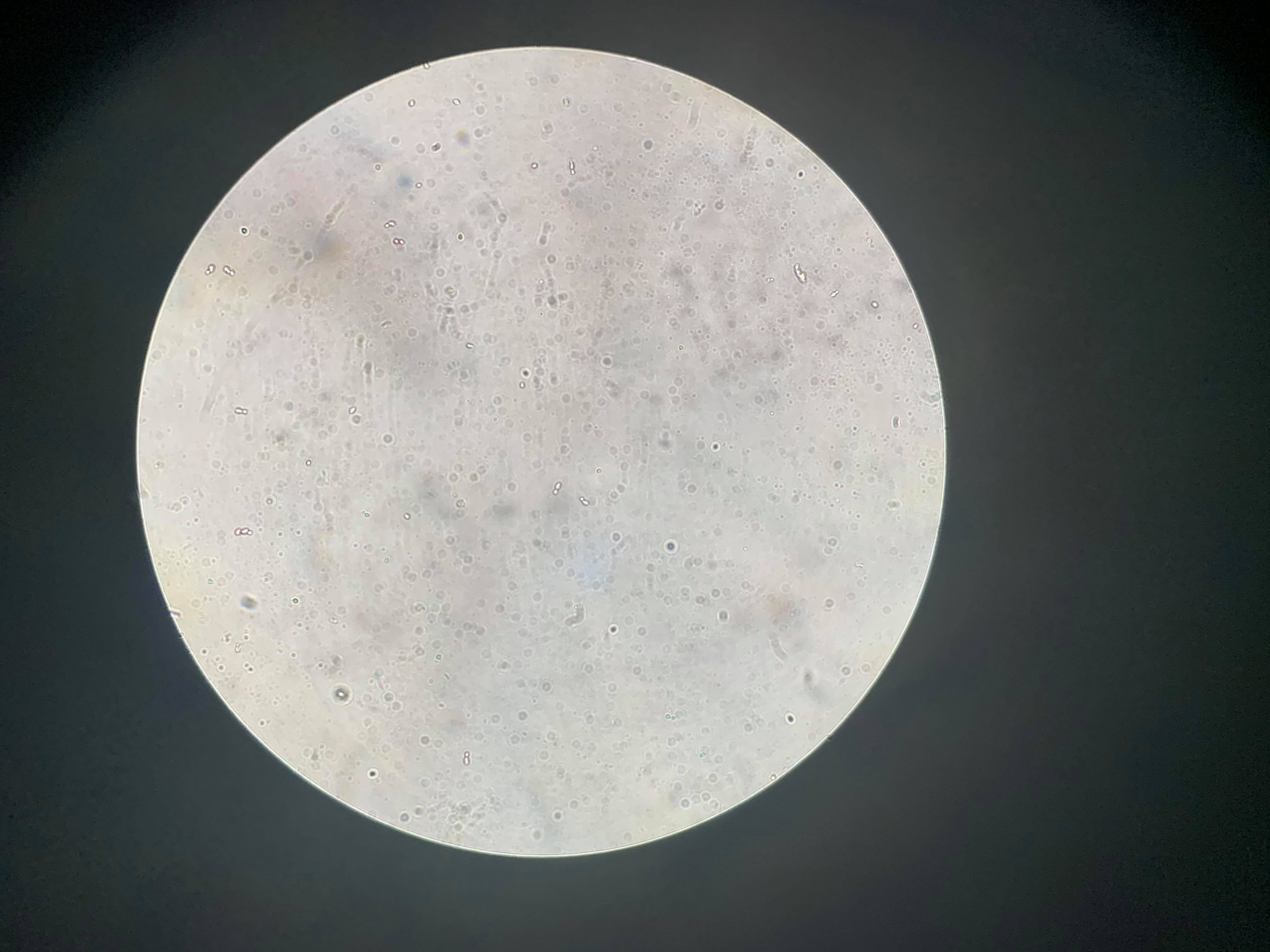
**

**Bright field**

**DAPI**

**PI**

Supplement: Supplementary Materials — Supplementary Table 1: parameters for EEP-NPs and polymer-NPs preparation. Supplementary Table 2: list and sequences of primers [71, 72]. Supplementary Figure 1: EEP-NP 2-inhibited C. albicans hyphal germination. Supplementary Figure 2: EEP-NP 2-induced cell death in C. albicans. [file 3715481.f1.zip › Supplementary Figure 2f_November 16, 2019_ECAM_2932134.docx]
